# Epigenetic targeting of MECOM/KRAS axis by JIB-04 impairs tumorigenesis and cisplatin resistance in MECOM-amplified ovarian cancer

**DOI:** 10.1038/s41420-025-02618-2

**Published:** 2025-07-15

**Authors:** Ibha Singh, Amarnath Karna, Anita Prajapati, Ujjawal Solanki, Archana Mukherjee, Sheetal Uppal, Pawan Malhotra, Manoj Kumar, Pallavi Agarwal

**Affiliations:** 1 Amity Institute of Molecular Medicine and Stem Cell Research, Amity University Uttar Pradesh, Noida, India; 2 Amity Institute of Genome Engineering, Amity University Uttar Pradesh, Noida, India; 3 Radiopharmaceuticals Division, Bhabha Atomic Research Centre, Mumbai, India; 4 Molecular Biology Division, Bhabha Atomic Research Centre, Mumbai, India; 5 International Centre for Genetic Engineering and Biotechnology (ICGEB), Aruna Asaf Ali Marg, Jawaharlal Nehru University, Delhi, India

**Keywords:** Targeted therapies, Cancer genomics

## Abstract

Copy number gene amplification and associated overexpression of driver oncogenes are genetic events that contribute to cancer progression and drug resistance. MDS1 and EVI1 Complex locus (*MECOM*) gene is copy number amplified and overexpressed in aggressive epithelial ovarian cancers. The biological function and precise molecular mechanism of MECOM in the progression and drug resistance of ovarian cancer remain unclear. Here, we unravel MECOM as a regulator of *KRAS* and its downstream MAP Kinase signalling pathway, and also identify epigenetic inhibitor JIB-04 as a pharmacological agent targeting MECOM/KRAS axis. RNAi-mediated attenuation of *MECOM* in ovarian cancer cells harboring *MECOM* amplification reduced their proliferation, impaired colony formation, and impeded cellular migration. ChIP-qPCR analysis confirmed binding of MECOM to the KRAS promoter, suggesting direct regulation of the *KRAS* gene at the transcriptional level. Further, MECOM promoted cellular proliferation by regulating *KRAS-*mediated ERK/ZEB1 signalling cascade. The anti-tumorigenic effects due to MECOM loss were phenocopied by the treatment of ovarian cancer cells harboring *MECOM* amplification with JIB-04 epigenetic inhibitor targeting Jumonji domain histone demethylase enzymes. By ChIP-qPCR, we show that JIB-04 induced transcriptional changes of MECOM by altering H3K27me3 demethylation at its promoter region. We further report that ovarian cancer cells expressing high-MECOM levels exhibit cisplatin resistance, which could be effectively reversed upon pre-treatment with JIB-04. The therapeutic efficacy of JIB-04 was further demonstrated in mice bearing ovarian cancer cell xenografts, where JIB-04 slowed down the tumor growth in corroboration with diminishing *MECOM* expression. RNA-sequencing analysis identified potential cisplatin resistance gene, *SUB1*, being regulated by JIB-04-mediated modulation of MECOM expression. Altogether, these data suggest that epigenetic silencing of MECOM by JIB-04 mediated H3K27me3 modulation is an important mechanism in ovarian cancer and provide a new therapeutic target for the treatment of ovarian cancers harboring MECOM amplification.

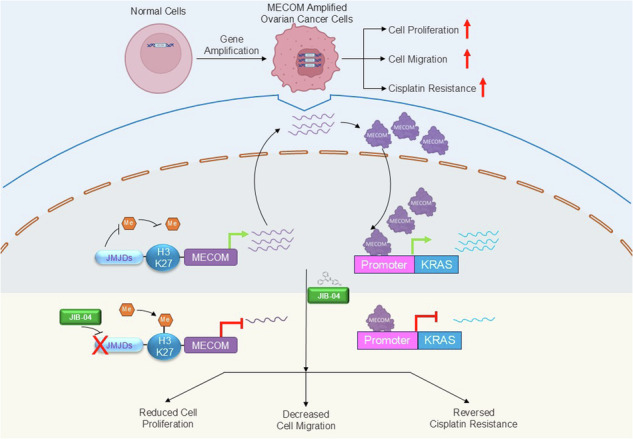

## Introduction

Ovarian cancer ranks third among female reproductive cancers with global incidence rate of 6.6 per 100,000 and a mortality rate of 3.9 per 100,000 [1, 2]. Patients respond well to cisplatin-based chemotherapy; however, within a period of 6–12 months, they develop drug resistance, contributing to poor 5-year relapse-free survival rate of 46% [2]. Amid several molecular events causative of drug resistance in ovarian cancer, more than 10% cases of ovarian cancer patients (*n* = 489) exhibit copy number amplification of oncogenes such as cyclin E1 (*CCNE1)*, Kirsten-rat sarcoma virus (*KRAS)*, MDS1–EVI1 complex locus (*MECOM*), and Mitogen-activated protein kinase 1 (*MAPK1*) exemplifying their potential as therapeutic targets [3–5]. Consequently, targeting gene copy number amplification in drug resistant ovarian tumors represents a potential therapeutic strategy to improve patient treatment and management.

Among the above amplified oncogenes, *MECOM* is a complex unified transcriptional locus, consisting of two promoters driving expression of MDS1 and EVI1 genes, and transcribing two major splice variants i.e., MDS1–EVI1 (Myelodysplasia Syndrome 1-Ecotropic Virus Integration site 1 protein homolog) and EVI1 (Ecotropic Virus Integration site 1 protein homolog) [6–8]. MDS1–EVI1 is a result of splicing event from exon 2 of MDS1 to exon 2 of EVI1, and this splicing event occurs frequently in ovarian cancer [9]. Copy number amplification of *MECOM* locus is associated with elevated transcript and protein levels of both MECOM isoforms MDS1–EVI1 and EVI1, in advanced ovarian cancers [10]. The Cancer Genome Atlas (TCGA) indicates that in addition to ovarian cancer, MECOM is also copy-number amplified in >5% cases in various cancers. For example, in acute myeloid leukemia, MECOM gene fusion and amplification mark aggressive disease and poor survival rate [11]. Further, MECOM has been associated with aggressiveness of intrahepatic cholangiocarcinoma and also functions as prognostic biomarker in glioblastoma multiforme, lung squamous cell carcinoma, clear cell renal carcinoma, and lung adenocarcinoma [12, 13]. The EVI1 isoform of MECOM promotes proliferation and reduces apoptosis in hilar cholangiocarcinoma via PTEN/AKT signaling pathway [14]. MECOM enhances proliferation and invasion potential in colon cancer cells [15, 16] and head and neck squamous cell carcinoma [17] and also antagonizes transforming growth factor-β (TGF-β) mediated growth inhibition of hepatocellular carcinoma [18]. EVI1 isoform expression diminishes growth potential and stem-like property of breast cancer cells [19–23]. Further, CRISPR-mediated depletion of MECOM attenuated growth potential and stemness in lung squamous cell carcinoma [24]. In ovarian cancer, EVI1/MECOM drives disease progression by interacting with various genes such as PAX8 [9], TGF-β [25], PBK [26], and also regulating estrogen signaling [27]. Despite these insights, the precise mechanisms by which MECOM regulates proliferation and migration in ovarian cancer remain underexplored, and no specific inhibitors or targeted therapies for MECOM are currently available. Hence, characterizing the biological function of MECOM in ovarian cancer is critical to develop strategies for therapeutically targeting this amplified oncogene.

For oncogene-dependent cancers, targeting global epigenetic deregulation has emerged as a promising therapeutic approach. Epigenetic rewiring not only alters changes in oncogene expression via DNA/histone modifications, but also generates new gene expression programs that diminish oncogene dependency [28]. For amplified oncogenes, the histone H3K4/K9/K27 methylation/acetylation-mediated transcriptional regulation plays crucial role in regulating associated tumorigenic programs [29]. For example, balancing chromatin modifiers and histone-modifying enzymes controls EGFR amplification, demonstrating significant clinical implications of epigenetic therapies in cancer [30]. MYC transcription is regulated by histone acetylation in MYC amplified medulloblastoma patients, and BRD and HDAC inhibitors attenuated tumor growth in xenograft mice [31]. MAPKs (Mitogen-activated protein kinases) dependency is influenced by epigenetic plasticity in BRAF-mutated melanoma, thus, epigenetic inhibitors targeting distinct epigenetic states play significant role [32]. Another study reported downregulation of oncogenic Homeobox B and D clusters upon treatment of epigenetic inhibitor JIB-04 by alteration of global histone H3K27 methylation [33]. JIB-04 is a small-molecule inhibitor of Jumonji family histone demethylases, targeting JARID1A, JMJD2A-E, and JMJD3 enzymes. Further, evidence of attenuating function of JIB-04 in Temozolomide-resistant glioblastoma is suggestive of therapeutic potential of this epigenetic modulator towards drug-resistant cancers [34, 35]

The impact of epigenetic modulation on MECOM-driven oncogenesis in ovarian cancer remains unknown. Thus, unraveling contribution of MECOM and its epigenetic modulation in mediating tumor progression and cisplatin resistance in ovarian cancer needed further investigation. In this study, we report that MECOM promotes cellular proliferation and migration in ovarian cancer cells harboring *MECOM* amplification and pinpoint cisplatin resistance as an intrinsic property of MECOM-amplified ovarian cancer cells. We identify a novel downstream target of MECOM, which is responsible for its pro-proliferative and pro-migratory functions. We reveal that MECOM-mediated transcriptional regulation of KRAS activates KRAS/ERK/EGR1 signaling axis, contributing to MECOM’s oncogenic activity in MECOM-dependent ovarian cancers. Screening for potential epidrugs with MECOM-modulatory function identified E-JIB-04 as a potent inhibitor with growth inhibitory properties and the ability to enhance cisplatin sensitivity of ovarian cancer cells harboring MECOM amplification. We further characterized the anti-cancer efficacy of E-JIB-04 against MECOM*-*dependent ovarian cancer cells in vitro and in vivo. We report that E-JIB-04 impairs proliferation and migration of *MECOM*-amplified cells by epigenetically targeting MECOM. Transcriptome analysis revealed a set of genes regulated by JIB-04 by MECOM modulation. Thus, epigenetic targeting with E-JIB04 and modulating MECOM expression could have significant clinical implications for ovarian cancers.

## Results

### MECOM silencing led to defects in cell proliferation and migration in ovarian cancer cells harboring *MECOM* amplification

**Fig. 1:**
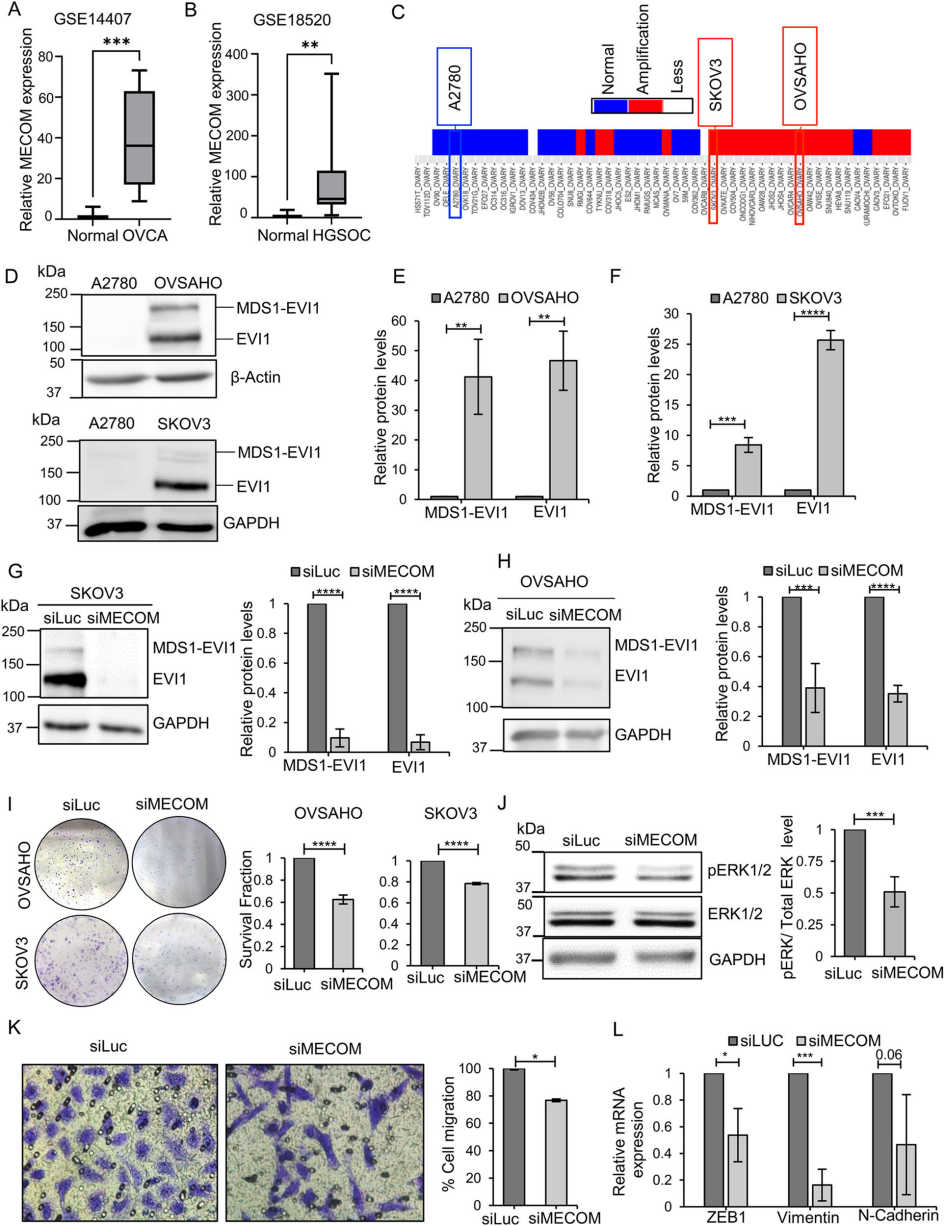
Knocking down MECOM in SKOV3 and OVSAHO ovarian cancer cells harboring MECOM amplification results in proliferation and migration defect. **A** High MECOM expression was observed in ovarian cancer patient samples (Tumor; *n* = 12) when compared to ovarian surface epithelium tissue samples (Normal; *n* = 12) (*p* value < 0.0001) **B** Tumor tissues of high grade serous ovarian carcinoma patients (HGSOC; *n* = 53) express high MECOM compared to normal ovarian surface epithelium tissues (Normal; *n* = 10) (*p* value < 0.001). **C** Selection of cell lines OVSAHO and SKOV3 with MECOM amplification, and non-amplified cell A2780 for the study based on Cancer Cell Line Encyclopedia (CCLE) dataset. **D**, **E**, **F** Western blotting demonstrated high protein expression of MECOM (MDS1-EVI1) and EVI1 in OVSAHO and SKOV3 ovarian cancer cells in comparison to A2780 non-amplified cells. Bar graphs provide quantification of protein bands analysed by densitometric analysis. **G**, **H** Western blotting and densitometric analysis confirmed efficient knockdown of MECOM in both SKOV3 and OVSAHO cells. siRNA targeting luciferase (siLuc) served as control. **I** Colony forming assay demonstrated proliferation defect in both cell lines upon MECOM silencing. **J** MECOM silencing reduced phosphorylation of ERK1/2 in SKOV3 cells. Bar graph provides the quantification of densitometric analysis of protein bands. **K** Transwell migration assay showed defective migratory potential of MECOM deficient cells **L** RT-qPCR analysis for ZEB1, Vimentin, and N-cadherin in SKOV3 cells upon MECOM silencing. Uncropped western blot images corresponding to Fig. 1G, H, and J were shown in Supplementary information.

MECOM amplification was analysed in publicly available pan-cancer TCGA genomic database, which revealed more than 20% copy number amplification of *MECOM* in ovarian epithelial tumors, only second to esophageal squamous cell cancers (https://www.cancer.gov/tcga) (Supplementary Fig. 1A) [36, 37]. The copy number amplification coincided with higher MECOM expression in patients of ovarian cancer compared to healthy controls as analysed by pan-cancer microarray datasets HG-U133 microarray (GPL570 platform) [38] (Supplementary Fig. 1B). Further, we evaluated MECOM expression in two independent patient datasets comprising of tumor samples from ovarian cancer patients and ovarian tissues from healthy volunteers. Notably, expression analysis of MECOM in GSE14407 dataset [39] showed significantly higher MECOM expression in ovarian cancer patient samples (*n* = 12) in comparison to normal ovarian surface epithelium samples (*n* = 12) (Fig. 1A). The GSE18520 dataset [40] compared gene expression of patients with high grade serous ovarian cancer (HGSOC) (*n* = 53) and matched controls (*n* = 10) and mean MECOM gene expression in these samples were analysed. We find that MECOM is also overexpressed in HGSOC cancer patients compared to normal ovarian tissue (Fig. 1B).

To unravel oncogenic function of MECOM, we selected OVSAHO and SKOV3 as ovarian cancer cell lines harboring high *MECOM* copy number amplification, and A2780 was selected as cell line lacking *MECOM* amplification (Fig. 1C). Compared to A2780 cells, OVSAHO and SKOV3 cells overexpressed both MECOM isoforms i.e., MECOM and EVI1 at protein level as confirmed by Western blotting (Fig. 1D, E, F). Loss of MECOM experiment was performed to understand its oncogenic function in SKOV3 and OVSAHO ovarian cancer cells that harbor MECOM amplification. Knockdown of *MECOM* using siRNA achieved ~70% reduction in both MECOM and EVI1 (Fig. 1G, H). *MECOM* silencing led to proliferation defect in cells with MECOM amplification as observed by reduced colony formation (Fig. 1I). To understand the molecular basis of this defect, we investigated Mitogen-activated protein kinases (MAPK) pathway, which majorly regulates cellular proliferation [41–43]. We observed reduced phosphorylation of Extracellular signal-regulated kinase 1/2 (ERK1/2) in MECOM-deficient cells, suggesting that MECOM promotes cellular proliferation in ERK1/2-dependent manner (Fig. 1J). Notably, *MECOM* silencing in SKOV3 cells resulted in migration defect (Fig. 1K), which may be mediated by MAPK/ERK1/2 regulated EMT pathway. Towards this end, we analysed levels of ZEB1, a critical mediator of epithelial-mesenchymal transition (EMT), which was found to be downregulated in MECOM-deficient cells. We further observed downregulation of N-cadherin, non-canonical downstream target of ZEB-1 (Fig. 1L), suggesting a potential transcriptional regulation of N-cadherin by ZEB-1 [44, 45]. Further, decreased expression of pro-migratory marker vimentin was observed in MECOM-deficient SKOV3 cells (Fig. 1K, L). Taken together, these findings establish MECOM as a growth-promoting and pro-migratory driver in cells harboring MECOM amplification.

### SKOV3 and OVSAHO ovarian cancer cells harboring MECOM amplification are sensitive to JIB-04, a histone demethylase inhibitor

**Fig. 2:**
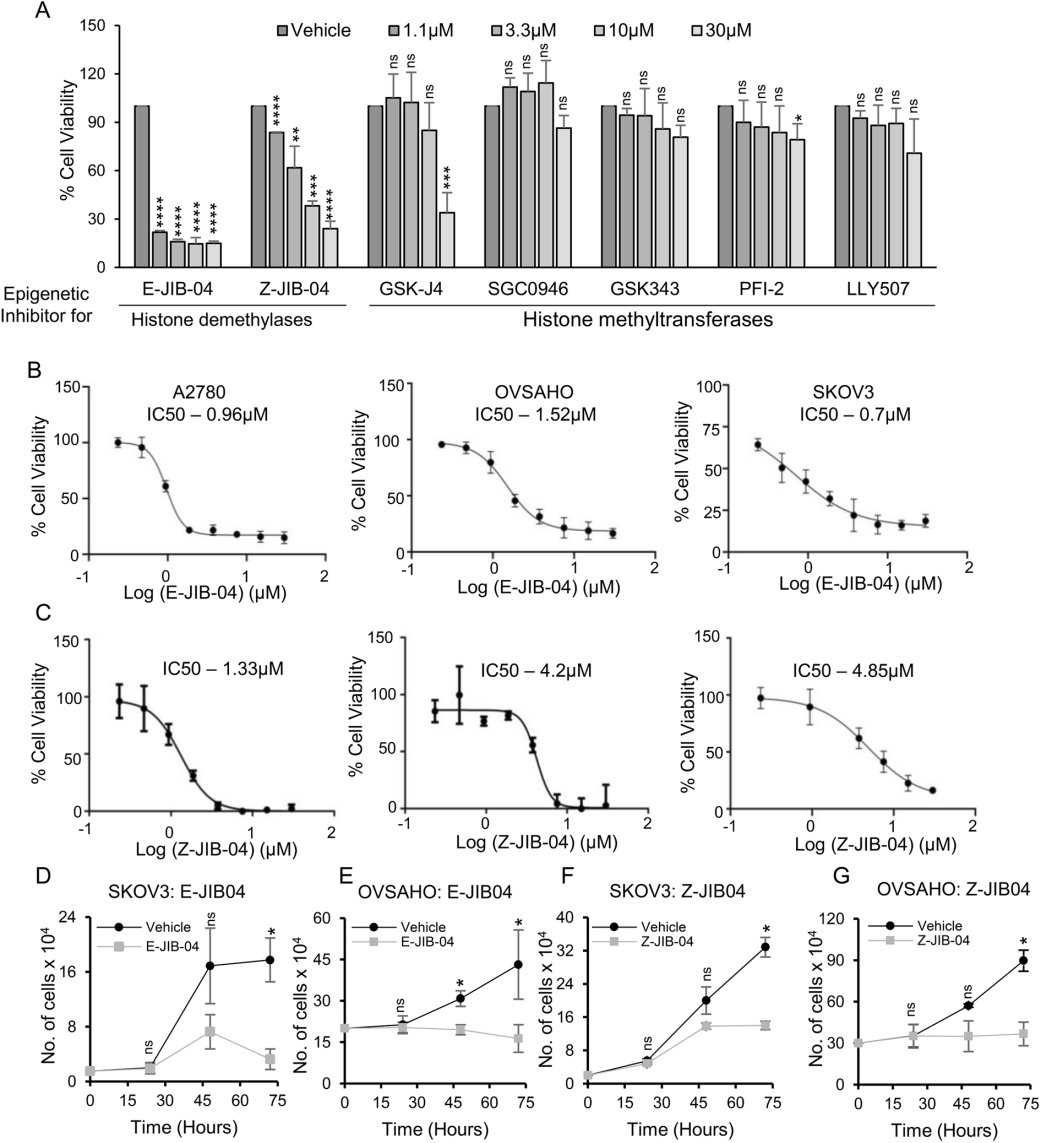
MECOM-amplified SKOV3 and OVSAHO ovarian cancer cells are vulnerable to epigenetic inhibitor E-JIB-04. **A** SKOV3 cells were treated with increasing concentrations (upto 30 µM) of epigenetic inhibitors targeting histone demethylases and methyltransferase enzymes, and the percentage cell viability was measured by crystal violet staining after 72 h. Quantitative analysis of percentage cell viability of A2780, SKOV3, and OVSAHO cells upon treatment with increasing doses of E-JIB-04 (**B**) and Z-JIB-04 (**C**). IC50 values demonstrate significant growth inhibitory effects on SKOV3 and OVSAHO by both E-JIB04 and Z-JIB04 isomers, showing better growth inhibitory effects than cisplatin (**D**–**G**). Treatment with both E and Z isomers of JIB-04 at IC50 values in SKOV3 and OVSAHO cells in a time-dependent manner suggested a proliferation defect.

MECOM displayed a cancer-promoting role in ovarian cancer cells with MECOM amplification, which intrigued us to look for MECOM inhibitors in the literature. We could not find any specific inhibitor targeting MECOM, hence, an alternative strategy to target tumor cells expressing high MECOM levels was envisaged. A study by Vazquez et al. reported epigenetic regulation of MECOM isoform EVI1 by histone modification in acute myeloid leukemia [46]. This prompted us to investigate whether MECOM transcription could be regulated by epigenetic mechanisms, specifically histone methylation in ovarian cancer. We screened a panel of epigenetic inhibitors targeting histone H3 methyltransferase and demethylases and assessed anti-tumorigenic effects of these epigenetic probes on ovarian cancer cells with MECOM amplification. Seven epigenetic probes (Supplementary Table 4), including 4 inhibitors targeting histone methyltransferases (DOT1L, EZH2, SETD7, SMYD2), while 3 inhibitors targeting histone demethylases (Jumonji KDM enzymes and UTX/JMJD3) were screened. Keeping highest concentration of 30 µM, a 3-fold dilution of the selected epigenetic probes was utilized to treat the SKOV3 cells for 72 h, and the cells were stained with crystal violet to measure the cell viability. Of all the inhibitors tested, both E and Z -isomers of JIB-04, inhibitor of pan-jumonji KDM enzymes, showed growth inhibitory effect at lower concentrations (Fig. 2A). Although Z isomer of JIB-04 is described as less potent than E-isomer [47], both isomers demonstrated dose dependent growth inhibitory effect in SKOV3 cells, hence both inhibitors were analysed for further experiments.

Cell viability assays were performed on A2780 (not expressing MECOM) and OVSAHO and SKOV3 (both possess MECOM overexpression and copy number amplification) to observe the growth inhibitory effects of E-JIB-04 and Z-JIB-04 (Fig. 2B, C). E-JIB-04 potently inhibited OVSAHO and SKOV3 cell growth at an IC50 values of 1.52 ± 0.057 µM and 0.7 ± 0.2 µM, respectively. The IC50 values for Z-JIB-04 treatment on OVSAHO and SKOV3 cells were 4.2 ± 0.08 µM and 4.85 ± 0.06 µM, respectively. We further tested both the isomers on non-tumorigenic immortalized BJ-TERT fibroblast cells and found that both inhibitors showed minimal toxicity to these cells at the tested concentrations (Supplementary Fig 2A).

We further analysed the effect of JIB-04 isomers on the proliferation rate of SKOV3 and OVSAHO cells by performing trypan blue exclusion assay in a time-dependent manner. Within a timeframe of 72 h, a significant abrogation of cellular proliferation was observed in response to JIB-04 inhibitors (Fig. 2D–G).

Collectively, these data implicate both the isomers of JIB-04 as potential inhibitors impeding growth of OVSAHO and SKOV3 cells harboring MECOM amplification, wherein the E-isomer demonstrated superior potency compared to Z-isomer. Notably, A2780 cells lacking MECOM amplification also displayed growth inhibitory effect in response to JIB-04 isomers, suggesting additional targets of these inhibitors.

### JIB-04 treatment mimics MECOM silencing by suppressing cancer phenotype via ERK/EGR1 and EMT pathway

**Fig. 3:**
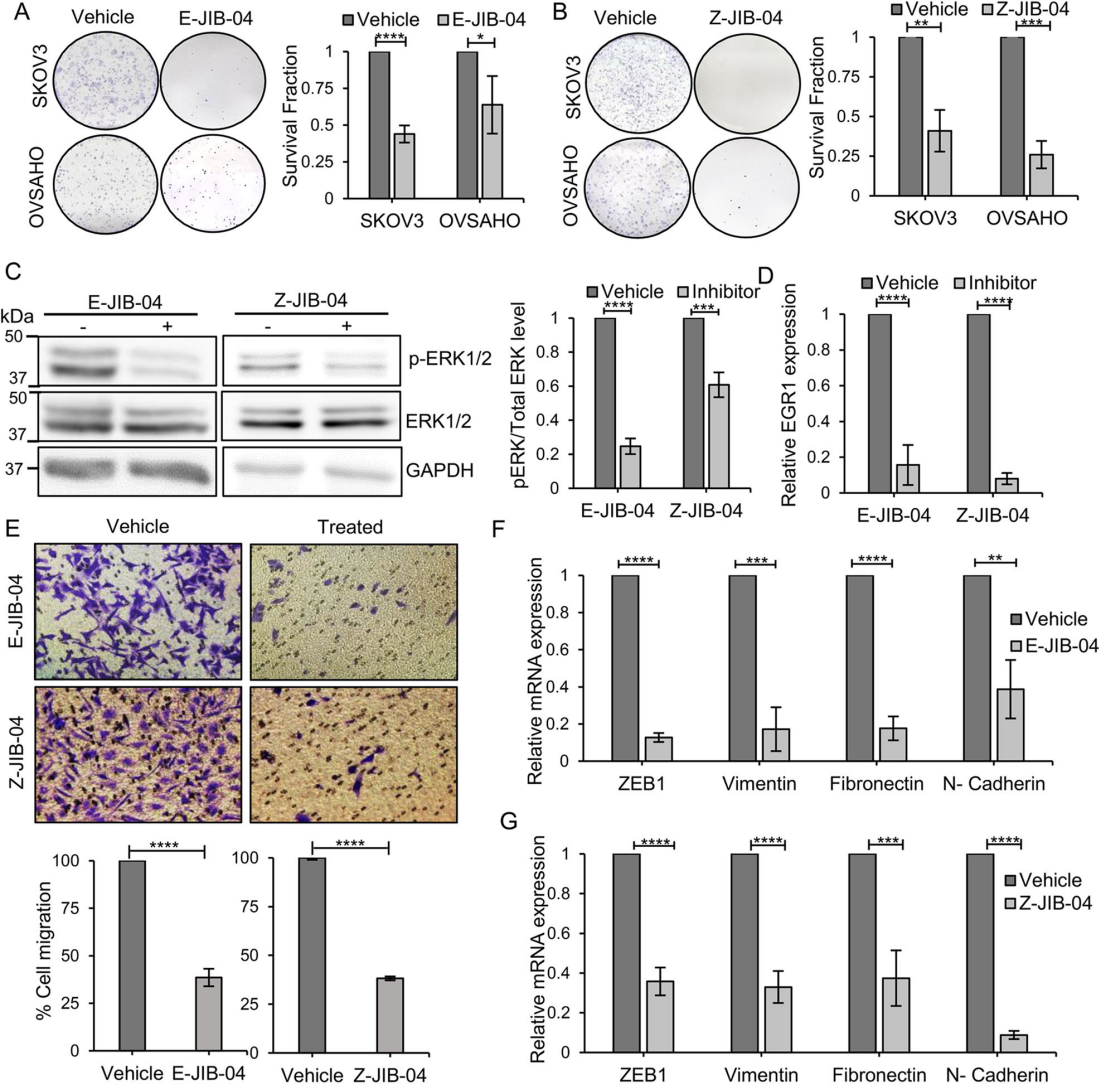
JIB-04 triggers proliferation and migration defects by modulating ERK-mediated signaling, thus phenocopying MECOM silencing. **A**, **B** MECOM-amplified SKOV3 and OVSAHO cells were treated with E-JIB-04 (0.7 µM; IC50) and Z-JIB-04 (6 µM; IC50) and were allowed to form colonies for 10 days. Significant reduction in survival fraction of treated cells was observed. **C** Western blot analysis resulted in reduction in phosphorylation of ERK1/2 upon treatment with E- and Z- forms of JIB-04 in SKOV3 cells. Bar graph provides the quantification of densitometric analysis of protein bands. **D** Realtime qPCR analysis revealed downregulation of EGR1, downstream signaling molecule of ERK in JIB-04 treated SKOV3 cells. **E** Inhibitory effect of E- and Z-forms of JIB-04 on SKOV3 cell migration using the transwell migration assay. Bar graph provides the quantification of percentage cell migration. Quantitative RT-qPCR of pro-migratory markers Vimentin, Fibronectin, N-cadherin, and EMT marker ZEB1 upon treatment of SKOV3 cells with E-JIB-04 (**F**) and Z-JIB-04 (**G**). Uncropped western blot images corresponding to Fig. 3C were shown in Supplementary information.

We further asked whether the anti-proliferative effects observed by JIB-04 treatment have some commonalities with MECOM silencing effects. We observed a consistent and significant decrease in number of colonies formed upon treatment with both isomers of JIB-04 on OVSAHO and SKOV3 cells (Fig. 3A, B). Further, this proliferation defect was contributed by impaired phosphorylation of ERK1/2 and downstream signaling (Fig. 3C), phenocopying the proliferation defects observed with MECOM silencing. One of the downstream targets of ERK1/2 is early growth response gene-1, *EGR1*, which promotes cellular proliferation by regulating cyclins and CDKs [48]. We also report a significant downregulation of EGR1 in response to JIB-04 inhibitors in ovarian cancer cells (Fig. 3D).

Given the migration defects observed in MECOM knockdown cells, we performed transwell migration assays in presence of both isomers of JIB-04. These experiments were performed only in SKOV3 as OVSAHO cells are nearly non-migratory in nature. Significant reduction in cellular migration was observed upon inhibitor treatment (Fig. 3E). The migratory defect after JIB-04 treatment was confirmed at the molecular level by discrete decrease in EMT-related transcription factor Zinc finger E-box binding homeobox 1 (ZEB1) and concomitant decrease in pro-migratory markers such as vimentin, fibronectin and N-cadherin [49] (Fig. 3F, G), which are reported earlier to be regulated by ZEB1 [50]. Thus, we identify that inhibiting histone demethylase enzymes using JIB-04 hinders tumorigenicity via mitigating ERK1/2-EGR1 mediated proliferation and ZEB1 driven EMT signaling pathways.

### JIB-04 treatment induces GADD45a/b mediated G2/M arrest and impairs mitotic events

**Fig. 4:**
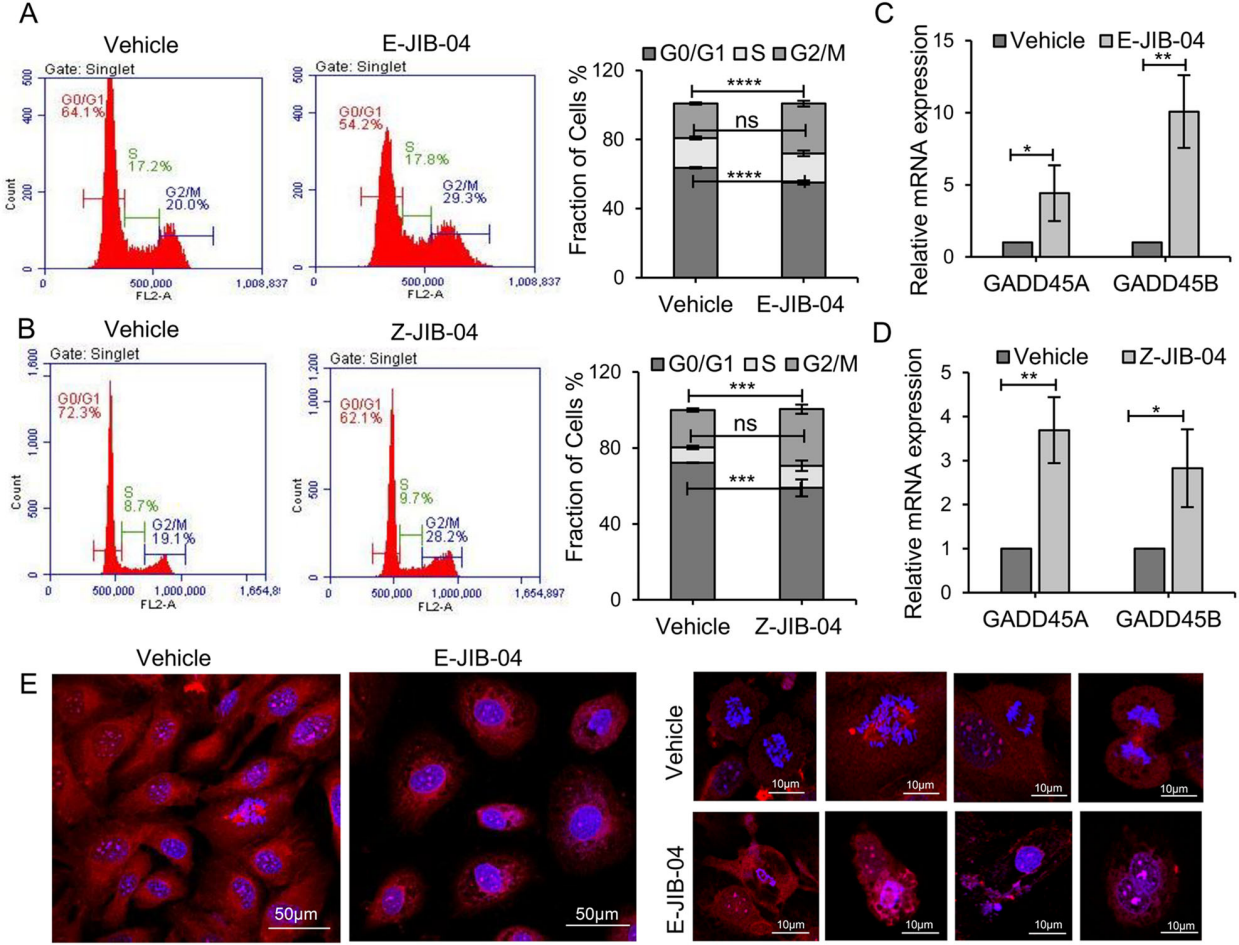
JIB-04 induces G2/M arrest via GADD45A/B modulation. SKOV3 cells were treated with E-JIB-04 **A** for 48 h and Z-JIB-04 **B** for 72 h, and the cell cycle was analysed by flow cytometry of propidium iodide-stained cells. Bar graph provides percentage of cells in G1, S, and G2/M phases. **C**, **D** Realtime qRT-PCR analysis revealed induction of GADD45A and GADD45B transcripts in SKOV3 cells in response to E- and Z-isomers of JIB-04 (**E**). Representative confocal fluorescent microscopy images of cells and their microtubule networks in control and E-JIB-04 (0.7 µM) treated SKOV3 cells post 72 h of inhibitor treatment. α-tubulin (red) and DAPI-stained nuclei(blue). E-JIB-04-treated cells showed abnormal cell mitosis compared to vehicle-treated cells.

To further understand the mechanism behind proliferation defects, we analysed cell cycle distribution in JIB-04 treated SKOV3 cells and observed induction of G2/M arrest **(**Fig. 4A, B). MAPK regulated genes GADD45a (growth arrest and DNA damage inducible 45 alpha) and GADD45b (growth arrest and DNA damage inducible 45 beta) regulate G2/M transition by inhibiting kinase activity of CDK1/cyclin B, the main regulators of G2/M transition [51–57] and hence we analysed the levels of these genes. We find that JIB-04 treatment significantly induced GADD45a and GADD45b transcript levels (Fig. 4C, D) thus contributing to G2/M arrest of JIB-04 treated cells. Further, the JIB-04-treated cells and corresponding control cells were immunostained with alpha-tubulin and nuclear stain 4′,6-diamidino-2-phenylindole (DAPI) to visualize the morphological defects induced by the inhibitor. Figure 4E shows the representative immunostained confocal images, where we observed that defective microtubule orientation, disrupted nuclei, and impaired mitosis persisted in JIB-04-treated cells, while normal mitotic events can be tracked optimally in vehicle-treated control cells.

### JIB-04 treatment regulates MECOM transcription by modulating H3K27 trimethylation and reduces ovarian tumor growth in xenograft mouse model

**Fig. 5:**
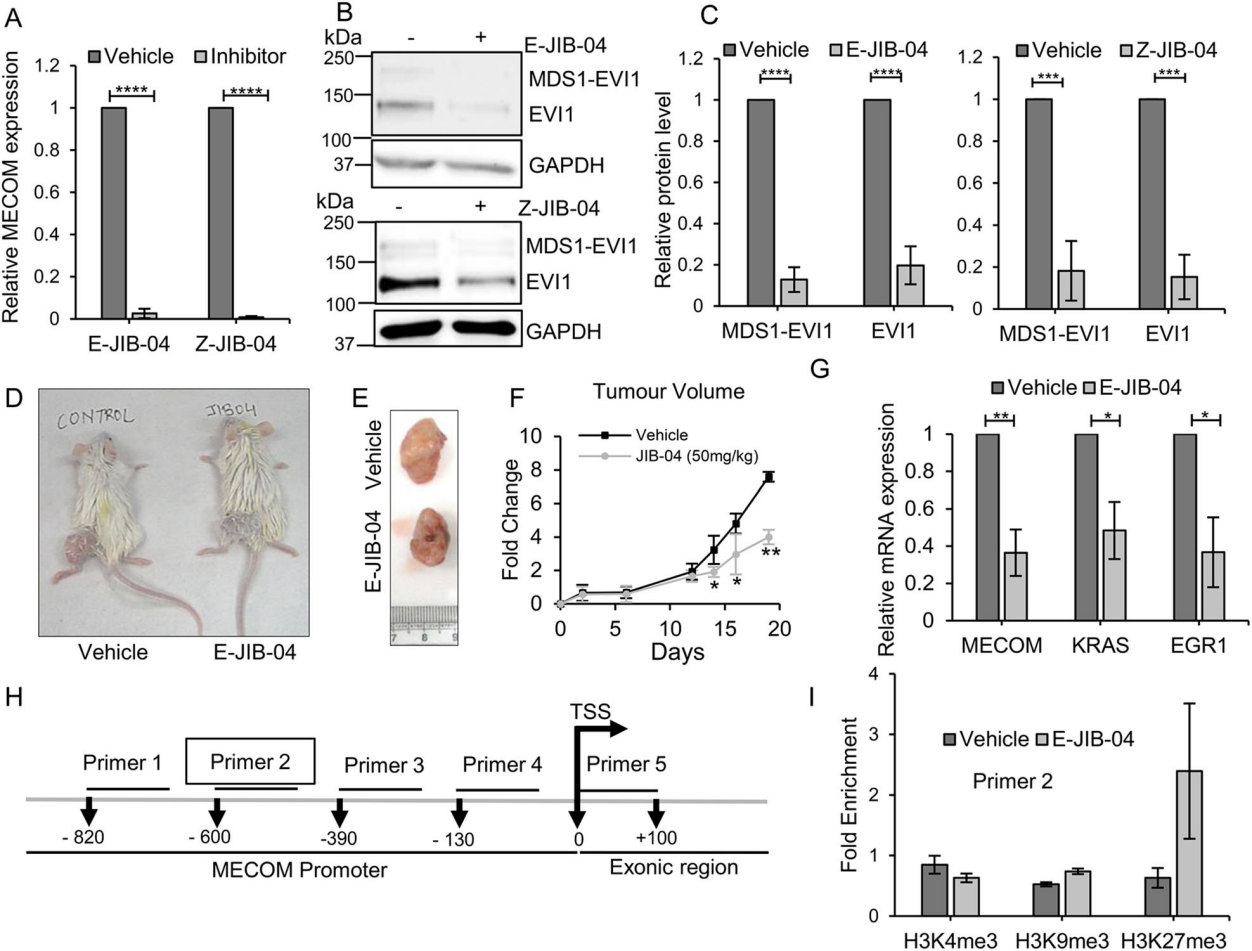
JIB-04 treatment modulates MECOM expression by altering H3K27me3 enrichment at its promoter and slows down tumor growth in mice xenograft model. Transcript (**A**) and protein **B**, **C** levels of MECOM (MDS1/EVI1) and EVI1 were analysed after E-JIB-04 (0.7 µM) and Z-JIB-04 (6 µM) treatment of selected MECOM-amplified cell lines using RT-qPCR and Western blotting. Bar graphs provide the protein quantification by densitometric analysis of protein bands. **D**, **E** Subcutaneous tumors generated from SKOV3 cells were allowed to reach a volume between 150 and 200 mm^3^, and then mice were treated every week with vehicle (*n* = 3) and E-JIB-04 50 mg/kg (*n* = 3) for a total of 3 doses. Xenografts in the E-JIB-04 treated mice were smaller than vehicle treated mice. **F** Reduction of tumor volume in E-JIB-04-treated mice was observed compared to the vehicle group. **G** RNA was extracted from tumors, and mRNA levels of MECOM, EGR1, and KRAS were measured by RT-qPCR. **H** Schematic representation of location of ChIP-qPCR primers on the promoter/exonic region of MECOM **I** Chromatin immunoprecipitation (ChIP) was performed using chromatin from SKOV3 cells treated with E-JIB-04 (0.7 µM). ChIP-qPCR was performed using antibodies targeting histone H3K4me3, H3K9me3, and H3K27me3, and qPCR was performed to check for enrichment of these histone marks on MECOM promoter region upon E-JIB-04 treatment. ChIP-qPCR using primer two showed increased H3K27me3 occupancy on MECOM promoter in the cells treated with the E-JIB-04 inhibitor. Uncropped western blot images corresponding to Fig. 5B were shown in Supplementary information.

Having observed that JIB-04 treatment phenocopied *MECOM* silencing, we asked whether JIB-04 treatment might regulate MECOM expression. Treatment with both E- and Z-JIB-04 isomers led to downregulation of MECOM at transcript and protein level (Fig. 5A–C). As JIB-04 treatment reduced MECOM expression in ovarian cells harboring MECOM amplification, we aimed to explore the tumor inhibitory activity of JIB-04 towards ovarian cancer cells harboring MECOM amplification, and efficacy studies were performed in SKOV3 cell-derived ovarian tumor mice model. E-JIB-04 isomer was injected intratumorally at 50 mg/kg, while the control group received vehicle only. The tumors were excised after treatment, and the volume of E-JIB-04-treated and vehicle-treated tumors was quantified. Representative photographic images of mice with tumors treated with E-JIB-04 and corresponding controls along with the respective excised tumors, are provided in Fig. 5D. We find that E-JIB-04 treatment reduced the tumor volume compared to vehicle-treated tumors (Fig. 5E, F). This in vivo effect of E-JIB-04 strengthens the value of this epigenetic inhibitor as a potential therapeutic molecule targeting ovarian cancer cells. Further, qRT-PCR analysis of extracted mRNA from the E-JIB04 and control-treated mice tumor tissues showed decrease in levels of MECOM (Fig. 5G). Analyzing the effect on cellular proliferation, we observed downregulation of EGR1 in JIB-04-treated mice tumors, as was seen in ovarian cancer cells (Fig. 5G). Further, considering that KRAS is upstream of MAPK proliferation signaling pathways, we analysed whether JIB-04 might affect KRAS levels. We find that KRAS transcript levels were downregulated in JIB-04-treated mice tumors (Fig. 5G).

Noticing MECOM transcript regulation by JIB-04 in cancer cells as well in tumors, we were intrigued to investigate JIB-04 mediated epigenetic regulation of MECOM by modulating histone modifications. Being an inhibitor of Jumonji family histone demethylase, JIB-04 treatment modulates methylation at histone H3 residues lysine 4, lysine 9, and lysine 27 (Supplementary Fig. 2B, C) [47]. To inquire for a potential epigenetic regulation of MECOM by histone methylation, we performed chromatin immunoprecipitation of ovarian cancer cells treated with or without E-JIB-04 inhibitor using ChIP-grade histone H3K4me3, H3K9me3, and H3K27me3 antibodies. Quantification of the amount of chromatin immunoprecipitated with anti-trimethyl Lys4, Lys9, and Lys27 antibodies showed enrichment of repressive mark H3K27me3 at MECOM promoter region 500–600 bp upstream of transcription start site (TSS) in E-JIB-04 treated cells compared to vehicle-treated cells (Fig. 5H–I). While no enrichment was observed for both repressive H3K9me3 and active H3K4me3 histone marks at the promoter region in response to JIB-04 (Fig. 5I). This suggests that MECOM promoter is regulated epigenetically by histone H3K27 trimethylation in SKOV3 cells, and the gene can be repressed by modulating histone modification using E-JIB-04 inhibitor.

Our ChIP-qPCR analysis revealed that MECOM promoter is responsive to epigenetic reprogramming mediated by histone methylation and can be targeted epigenetically. Taking together, our findings suggest that E-JIB-04 mediated epigenetic regulation of MECOM via H3K27me3 is one of the mechanisms contributing to its anti-neoplastic effects.

### MECOM controls KRAS transcription by binding to KRAS promoter and is responsive to inhibitory action of JIB-04

**Fig. 6:**
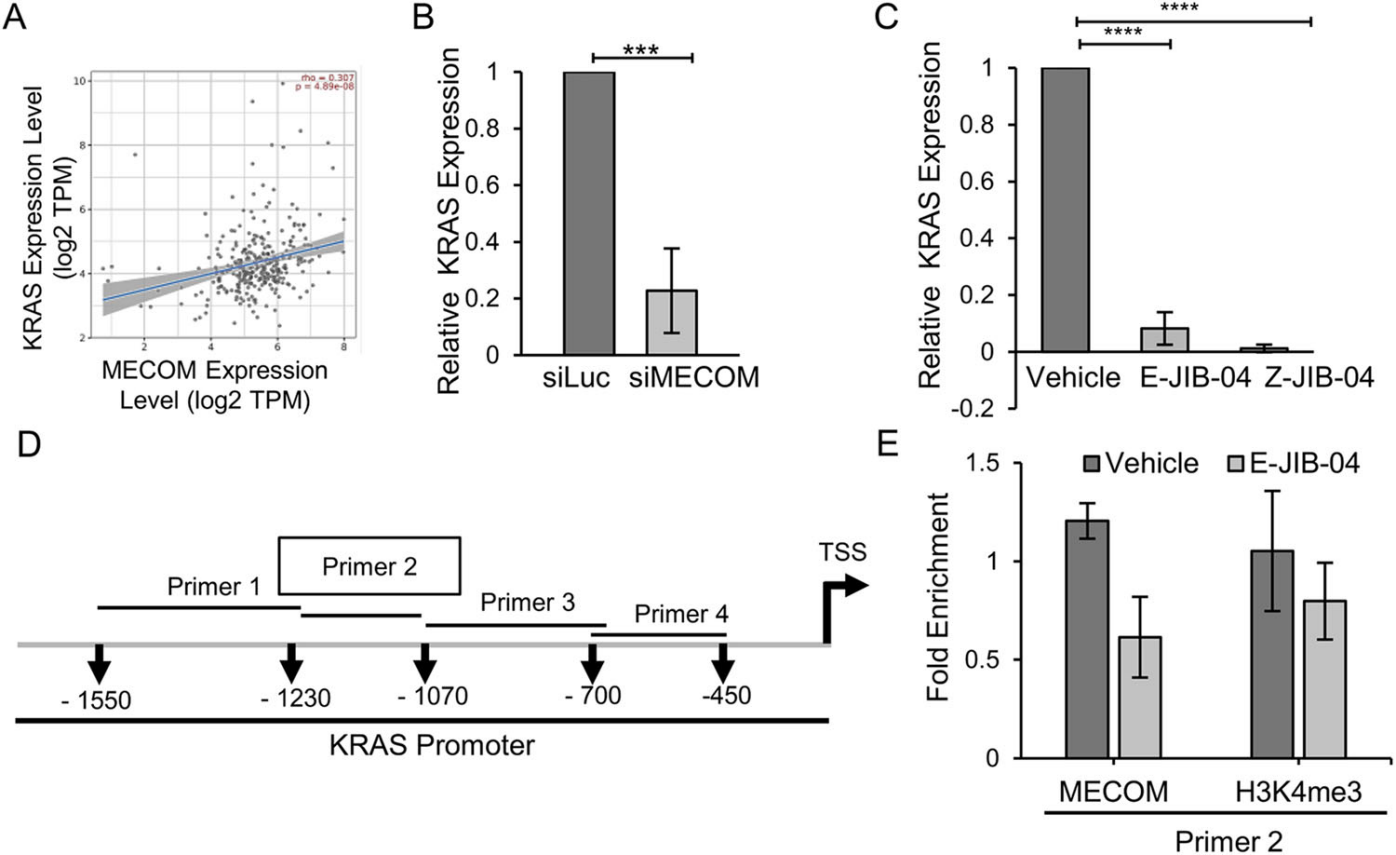
MECOM controls *KRAS* transcription by binding it’s promoter and is responsive to inhibitory action of JIB-04. **A** Significant positive correlation of MECOM with KRAS expression in ovarian cancer samples (*n* = 303). **B** RT-qPCR of KRAS in MECOM deficient SKOV3 cells. **C** RT-qPCR of KRAS in E-JIB-04 and Z-JIB-04 treated SKOV3 cells. **D** Schematic of ChIP-qPCR primers on KRAS promoter. **E** Chromatin immunoprecipitation-qPCR was performed using antibodies targeting MECOM and H3K4me3, and qPCR was performed to check for its binding on KRAS promoter region in presence or absence of E-JIB-04 treatment. ChIP-qPCR using primer 2 showed decreased occupancy of MECOM on *KRAS* promoter in E-JIB-04 treated cells compared to vehicle-treated cells.

As JIB-04 treatment and *MECOM* silencing altered MAPK signaling cascade, we hypothesized that these events might affect *KRAS*, the upstream component of the RAS/MAPK signaling pathway. In pancreatic cancer, correlation of MECOM and KRAS has been reported via microRNA-96 [58]. This further supported our hypothesis, and we first analysed the correlation of their expression using gene correlation module of TIMER2.0 webtool in publicly available ovarian cancer patient datasets (*n* = 303) [59]. A positive spearman’s rho value of 0.3 and a *p* value < 0.05 marked a positive gene expression correlation of MECOM and KRAS (Fig. 6A). By quantitative real-time qRT-PCR, we further confirmed a striking decrease in the transcript levels of *KRAS* upon MECOM siRNA-mediated silencing as well as in JIB-04-treated cells (Fig. 6B, C). A direct protein-DNA interaction of MECOM transcription factor on promoter region of *KRAS* gene was further established by ChIP-qPCR. Four ChIP-qPCR primers (Fig. 6D and Supplementary Table 3) covered the *KRAS* promoter region, and we find that MECOM enriched nearly 1000 bp upstream of TSS on KRAS promoter (Fig. 6E). This MECOM enrichment was reduced upon E-JIB-04 treatment, as MECOM is transcriptionally downregulated in response to epigenetic modulation of E-JIB-04 (refer Fig. 5A). A concomitant decrease in H3K4me3 at the same site further confirmed transcriptional repression of KRAS (Fig. 6E).

These results suggest MECOM as an upstream regulator of *KRAS* transcription. Thus, we propose that a primary mechanism underlying anti-proliferative action of JIB-04 on *MECOM* amplified cells is the inhibition of the MECOM/KRAS axis and its downstream signaling cascade.

### JIB-04 pre-treatment partially reverses cisplatin resistance of ovarian cancer cells harboring MECOM amplification

**Fig. 7:**
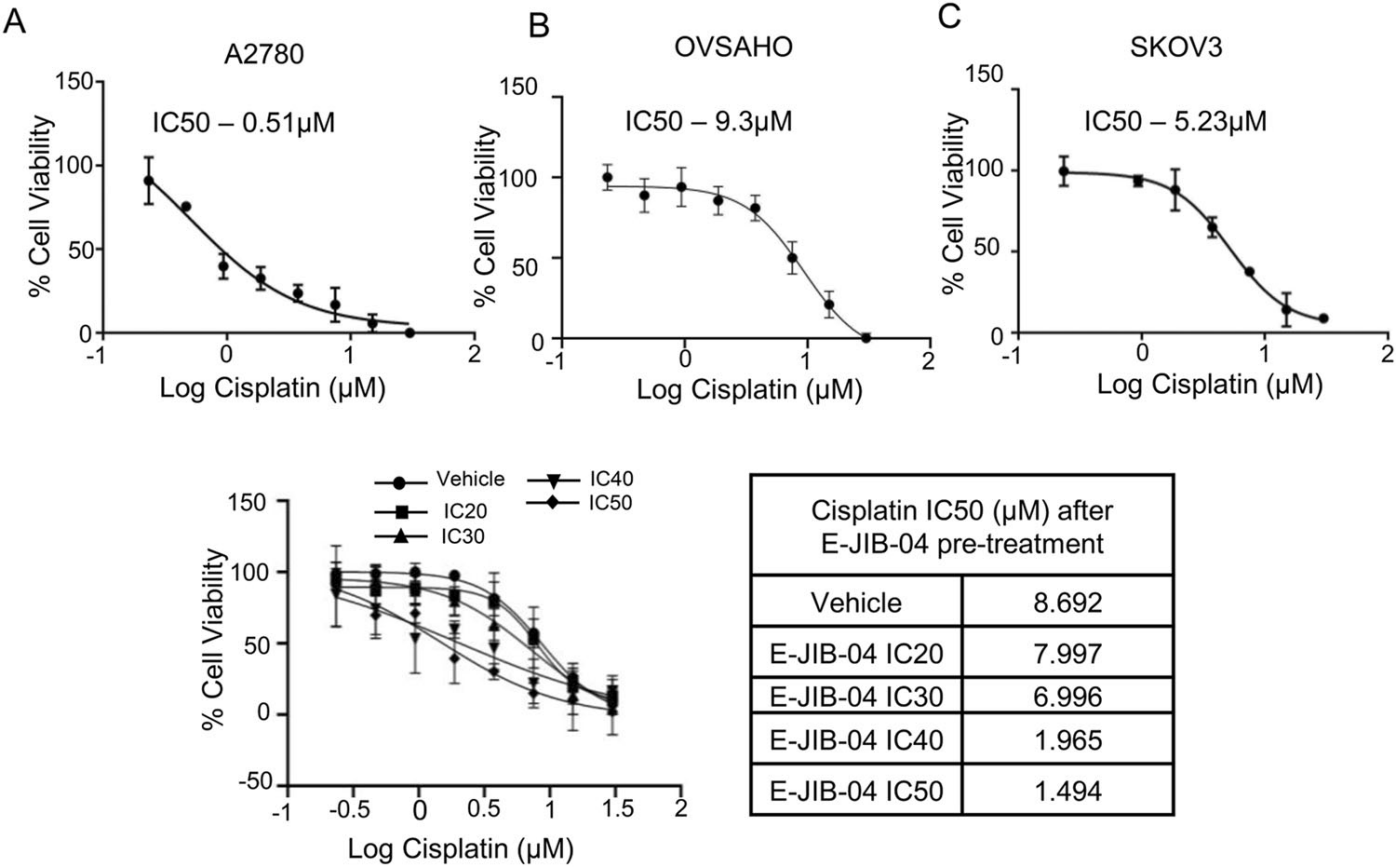
Ovarian cancer cells harboring MECOM amplification are resistant to cisplatin and gets resensitized to cisplatin upon JIB-04 pretreatment. **A**, **B**, **C** Dose dependent cell viability assay for OVSAHO, SKOV3, and A2780 in response to increasing doses of cisplatin. IC50 values demonstrate that MECOM amplified ovarian cancer cell lines exhibit higher resistance to cisplatin than non-amplified cells. **D** SKOV3 cells were pre-treated with E-JIB-04 with indicated doses (IC20, IC30, IC40, IC50) for 24 h and then treated with 2-fold serial dilution doses of cisplatin starting from 30 µM to 0.23 µM as end concentration. The table provides IC50 values calculated for each indicated treatments. All data are plotted as mean ± SD.

Oncogene amplification has been associated with drug resistance in the literature [60–62]. Having established oncogenic function of MECOM in ovarian cancer, we asked if MECOM copy number amplification and its high expression might correlate with drug resistance in tumor cells. We evaluated sensitivity of ovarian cancer cells harboring MECOM-amplification to cisplatin, the standard first line of treatment for ovarian cancer patients [63]. The growth inhibitory effect of cisplatin on OVSAHO, SKOV3, and A2780 cells was measured using crystal violet staining-based cell viability assay in a dose-dependent manner. The IC50 for cisplatin upon treatment of OVSAHO, SKOV3, and A2780 were calculated to be 9.3 ± 0.13 µM, 5.23 ± 0.04 µM, and 0.51 ± 0.03 µM, respectively (Fig. 7A–C). Thus, we find that MECOM amplified SKOV3 and OVSAHO cells were 10 and 18-fold resistant to cisplatin when compared to A2780 cells, which lacks *MECOM* gene amplification. Next, we inquired whether epigenetic modulation by histone demethylase inhibitor JIB-04 might facilitate better sensitization to cisplatin. To explore the effect of E-JIB-04 on cisplatin sensitivity, we pre-treated the SKOV3 cells with different concentrations of E-JIB-04 (IC20, IC30, IC40, and IC50) for 24 h, while the control cells were treated with 1% DMSO as vehicle. After 24 h, the cells were treated with cisplatin for 72 h in a 2-fold serially diluted manner starting from 30 µM. For each dose of E-JIB-04 used for pre-treatment, IC50 values were analysed for cisplatin-treated cells. Interestingly, with increasing concentration of E-JIB-04, the IC50 of cisplatin was reduced, suggesting an epigenetic modulation of cisplatin sensitivity (Fig. 7D). Pre-treatment with IC20 and IC30 of E-JIB-04 showed mild enhancement in cisplatin sensitivity, however, with IC40 and IC50, there was a remarkable enhancement in cisplatin sensitivity. Nearly 7-fold decrease in IC50 of cisplatin was observed with IC50 concentration of E-JIB-04. These results indicated that JIB-04 enhances cisplatin sensitivity via epigenetic modulation.

### RNA-sequencing identifies SUB1 as a JIB-04-regulated gene linked to cisplatin resistance

**Fig. 8:**
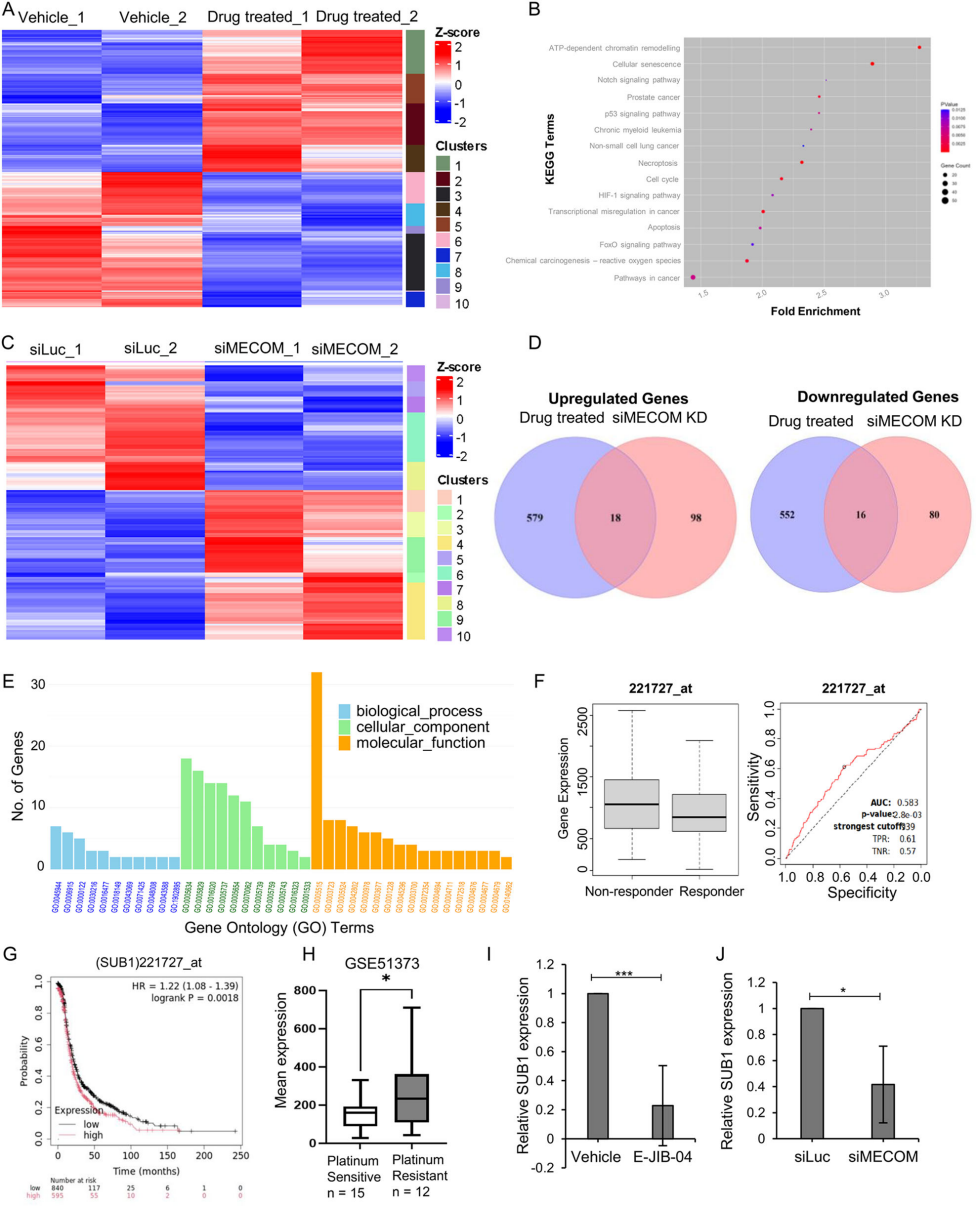
RNA-sequencing reveals potential cisplatin resistance genes regulated by MECOM dependency in ovarian cancer cells. **A** Heatmap of log2-fold change of the differentially expressed genes associated with JIB-04 exposure in ovarian cancer cells with MECOM amplification. **B** KEGG pathway analysis showing enrichment of cancer-associated genes upon JIB-04 treatment. **C** Heatmap of log2-fold change of DEGs associated with MECOM knockdown. **D** Venn diagrams displaying number of common genes differentially upregulated and downregulated in JIB-04 treated and MECOM-silenced ovarian cancer cells. **E** Gene ontology enrichment of 34 common DEGs in the biological process, cellular component, and molecular function **F** Box plot demonstrates correlation of SUB1 with platinum resistance. Ovarian cancer patient cohorts were treated with platin drugs and the response outcomes were determined by relapse-free survival over 6 months. SUB1 expression was significantly higher in non-responders to platin drug treatment (n = 114) when compared to responders (*n* = 1095); *p* value was 0.0035 as calculated by Mann–Whitney test. ROC plot illustrates the predictive biomarker value of SUB1 in response to platin therapy. **G** Kaplan Meier plot showing correlation of SUB1 expression with probability of progression-free survival of ovarian cancer patients (**H**). Mean SUB1 expression in tumor samples of platinum-sensitive (*n* = 15) and platinum resistant (*n* = 12) ovarian cancer patients as analysed in database GSE51373. Quantitative qRT-PCR confirms downregulation of SUB1 upon JIB-04 treatment (**I**) as well upon siRNA-mediated MECOM knockdown (**J**).

Observing that JIB-04 treatment resensitized ovarian cancer cells to cisplatin, we were intrigued to identify genes that might be associated with cisplatin resistance and transcriptionally regulated by JIB-04. To address this, we performed differential transcriptomic analysis of JIB-04-treated ovarian cancer cells harboring MECOM amplification along with respective control cells by RNA-sequencing. We identified 1165 differentially expressed genes in the drug-treated cells compared to vehicle control at a p-value of less than 0.05, as shown in the heatmap (Fig. 8A). As a proof of concept, MECOM gene fell in cluster 8, within the gene-sets downregulated upon JIB-04 treatment. Further, KEGG pathway analysis revealed enrichment of biological pathways such as transcriptional mis-regulation in cancer, multiple cancer pathways, cell cycle, apoptosis, necroptosis, p53, FoxO, Notch, and HIF1 signaling pathways in JIB-04 treated cells (Fig. 8B). To explore the role of MECOM in cisplatin resistance, we analysed global molecular changes accompanied with MECOM depletion by performing RNA-sequencing of MECOM knockdown cells and comparing with siLuciferase transfected cells. MECOM knockdown led to upregulation of 116 genes while 97 genes were downregulated as illustrated in heatmap (Fig. 8C). We then performed stringent filtering of expression data to only select for genes that exhibited overlap between the MECOM knockdown (KD) and JIB-04 treated cells and identified 34 differentially regulated genes (18 upregulated and 16 downregulated) overlapped between the two models (Fig. 8D and supplementary Tables 5 and 6). Further, gene ontology was performed to identify significantly affected pathways and molecular function for the common 34 differentially regulated genes (Fig. 8E and Supplementary Table 7). The biological processes mainly affected were cell migration, apoptosis, regulation of transcription, regulation of programmed cell death. Genes associated with cellular components were predominantly located in nucleus, mitochondria, membranes, and cytosol. While the molecular functions were most often related to DNA binding, transcription factor binding, RNA polymerase II cis-regulatory region sequence-specific DNA binding, and cadherin binding.

To identify relevant genes that are targeted by JIB-04 and MECOM modulation, we focussed on overlapping set of 34 genes that were differentially regulated both upon MECOM knockdown and JIB-04 treatment. We analysed publicly available ovarian cancer patient datasets, deciphering the potential correlation of expression of these 16 candidate genes with relapse-free survival (RFS > 6 months) in ovarian cancer patients treated with platinum-based drugs. The platin treated patient cohorts were subdivided into responders (RFS > 6 months; *n* = 1095) and non-responders (RFS < 6 months; *n* = 114) post-chemotherapy. Also, Receiver operating curve (ROC) plots analysed the clinical value of gene expression correlation as a predictor of non-responsiveness to drug treatment. For shortlisting relevant genes, we focussed on genes that showed an area under curve (AUC) > 0.55 in ROC plot analysis. Out of the 34 overlapping genes, 9 genes showed an AUC value of more than 0.55 in ROC analysis (Fig. 8F and Supplementary Fig. 3A–H). The next shortlisting of relevant genes was based on the correlation of high expression of these candidate nine genes with non-responsiveness to drug treatment. Only two genes SUB1 Regulator Of Transcription (SUB1) and ATPase family AAA domain-containing protein 2 (ATAD2) qualified this shortlisting, and both were downregulated by JIB-04 treatment and upon MECOM silencing. Out of both of these genes, SUB1 expression was significantly higher in non-responders to platinum drug based therapy (Mann–Whitney test; *p* value = 0.0035) (Fig. 8F). An AUC value of 0.58 (*p* value = 2.8e-03) suggested a potential of SUB1 as a predictive biomarker of clinical utility to predict relapse free survival of ovarian cancer patients in response to platin therapy.

Considering a better AUC for SUB1, we analysed the prognostic significance of SUB1 (Probe id = 221727_at) using Kaplan Meier survival plot, determining the impact of SUB1 expression on progression-free survival (PFS) of ovarian cancer patients. Patients with higher SUB1 expression exhibited a poorer progression-free survival compared to those with lower SUB1 expression (Fig. 8G). In an independent publicly available database (GSE51373) encompassing both chemotherapy-sensitive (PFS > 18 months) and -resistant (PFS < 8 months) HGSOC patients, we observed significantly higher SUB1 expression in tumor samples of platinum-resistant HGSOC patients compared to sensitive ones (*p* < 0.01) (Fig. 8H). Further, qRT-PCR analysis confirmed that JIB-04 treatment and MECOM siRNA-mediated silencing reduced SUB1 transcript levels in ovarian tumor cells (Fig. 8I, J). These results indicate that SUB1 is a potential prognostic marker predicting platinum drug resistance. Further, SUB1 expression is regulated epigenetically by JIB-04, most likely by modulating MECOM transcriptional activity.

In totality, our results thus present a new epigenetic based therapeutic opportunity to target ovarian cancer cells harboring MECOM amplification and may also suggest potential of combining JIB-04 epidrug as neo-adjuvant with standard platin based chemotherapy.

## Discussion

Gene amplification is a frequent chromosomal abnormality associated with carcinogenesis and malignant phenotypes. In this study, we identified the oncogenic function of MECOM gene in ovarian cancer cells harboring *MECOM* copy number amplification. The selected amplified ovarian cancer cells exhibited MECOM protein overexpression, and abrogating endogenous MECOM expression in these cells disrupted key cancer characteristics, including enhanced proliferation and migration. We demonstrate that MECOM promotes proliferation and migration of MECOM-amplified ovarian tumor cells by regulating KRAS/pERK1/2/ ZEB1 signaling cascade. Our study in ovarian cancer epithelial cells consistently showed that MECOM loss, as well as JIB-04 treatment, downregulated ZEB1 along with its non-canonical targets, N-cadherin and vimentin, suggesting potential transcriptional regulation of N-cadherin and vimentin. Our findings corroborate with previous reports demonstrating ZEB1-mediated regulation of N-cadherin in prostate cancer cells and vimentin in melanoma [44, 45, 64]. Further, ZEB1-silenced human esophageal epithelial cells resulted in transcriptional downregulation of N-cadherin and vimentin compared to control cells [65]. These reports reinforce our observation of ZEB1-driven transcriptional control of N-cadherin and Vimentin in ovarian cancer. Future investigations are warranted to elucidate how MECOM loss or epigenetic inhibitors, such as JIB-04, modulate ZEB1’s transcriptional activity on canonical target gene promoters, further clarifying the molecular underpinnings of this oncogenic axis.

MECOM is a zinc finger transcription factor regulating critical physiological processes such as cell proliferation, differentiation, haematopoiesis, and also regulates gene transcription [8, 66]. MECOM isoform EVI1 has been reported to directly regulate gene transcription of MS4A3 in myeloid leukemia [67] and of GATA2, regulating hematopoietic stem cell proliferation [68]. In colorectal cancer, MECOM preferentially activates the proto-oncogene ETS2 by binding to its distal super-enhancer [69]. An indirect regulation of KRAS oncogene via MECOM by modulating miRNAs such as miRNA-143 [70] and miRNA-96 [58] is also reported. Here, our results unveil a previously undescribed role for MECOM in the transcriptional regulation of KRAS oncogene at chromatin level, consolidating its importance as a relevant target for therapeutic intervention in ovarian cancer. Notably, prior research has shown that MECOM binds to *VEGFR2* promoter with a consensus nucleotide sequence of [AG]TGA[CG]T[CA]A [71]. Interestingly, we find a potential MECOM-binding sequence, “ATGACTC,” within the 1 kb upstream region of the KRAS transcription start site, where MECOM enrichment occurs. Further studies are required to precisely pinpoint the exact consensus sequence for MECOM with which it binds to KRAS promoter, enhancing our understanding of its transcriptional regulatory mechanisms.

A strategy to target amplified driver oncogenes is to identify epigenetic mechanisms that might modulate their expression and thus their tumorigenic effects. Towards this end, we identified JIB-04, a Jumonji-domain histone demethylase inhibitor, as a potential epigenetic targeted therapy for ovarian cancers harboring *MECOM* amplification. Although our epigenetic screening revealed E-and Z-isomers of JIB-04 as potent inhibitors for *MECOM* amplified cells, E-JIB-04 exhibited superior anti-tumor activity at a comparatively lower concentration; hence, we propose E-isomer of JIB-04 as a preferred epidrug. JIB-04 treatment suppressed cell proliferation by altering KRAS-ERK1/2 signaling axis, similar to MECOM silencing. The inhibitor treatment also induced G2M arrest concomitant with enhanced GADD45A and GADD45B transcription. A key mechanism underlying these anti-cancer effects was JIB-04’s epigenetic modulation of MECOM via altering H3K27me3 deposition at its promoter. This gene-specific epigenetic regulation parallels previous findings, where JIB-04 modulated AKT2 transcription through H3K27me3 [72], reinforcing its potential as a targeted therapeutic for MECOM-driven ovarian cancers.

Earlier, therapeutic activity of JIB-04 has been demonstrated against drug resistance in glioblastoma cells [35] and radio resistance in NSCLC [73]. Another study reported that JIB-04 targeted imatinib-resistant chronic myeloid leukemia (CML) cells by enriching H3K36me3 on tumor suppressor gene SETD2 promoter and enhancing its transcription [74]. In our study, ovarian cancer cells harboring MECOM amplification showed higher cisplatin resistance compared to cells lacking amplification. Notably, pre-treatment with JIB-04 improved cisplatin sensitivity of *MECOM* amplified ovarian cancer cells. Thus, our findings, along with previous reports, indicate JIB-04 as an epigenetic inhibitor that may act as an adjuvant with various chemotherapies or serve as a neo-adjuvant, warranting further in-depth exploration. Upon inquiring for potential genes contributing to cisplatin resistance due to MECOM amplification, we identified transcription co-activator SUB1, a proposed carcinogenic gene implicated in hepatoblastoma malignancy [75] and associated with poor prognosis in breast cancer metastasis [76]. Our study is the first to propose SUB1 as a potential predictive gene for drug resistance, and future studies will determine the exact mechanistic function of SUB1 in drug-resistant pathways.

JIB-04 demonstrates potent anti-tumorigenic properties across multiple in vivo studies [47, 73, 77, 78]. It effectively suppresses lung cancer progression, extends survival in tumor-bearing mice, and enhances radiosensitivity [73]. By targeting KDM5B and HSP90, JIB-04 overcomes chemoresistance in gastric cancer [77]. Our findings reveal that JIB-04 exerts anti-tumorigenic effects in ovarian cancer cell-derived mouse xenografts by inhibiting MECOM/KRAS-mediated proliferation signaling. Notably, JIB-04 modulates ZEB1, a key regulator of epithelial-mesenchymal transition (EMT), which drives aggressive traits in ovarian cancer, including invasion and metastasis. This observation underscores the need for in vivo studies to elucidate the metastatic impact of this epigenetic inhibitor. Large-scale, rigorous investigations in animal models and patient-derived samples are essential to establish JIB-04 as a leading epidrug. Additionally, we demonstrate the ability of JIB-04 to sensitize MECOM-amplified ovarian cancer cells to cisplatin. Exploring the synergistic potential of JIB-04 and cisplatin in cisplatin-resistant ovarian cancer models holds significant promise. Future in vivo studies will prioritize combining JIB-04 with cisplatin to evaluate its efficacy as a transformative adjuvant along with standard chemotherapy.

Our study establishes the epidrug JIB-04 as a promising therapeutic agent for targeting the amplified oncogene MECOM in ovarian cancer. JIB-04 represses MECOM expression through H3K27me3-mediated epigenetic regulation, driving potent anti-tumorigenic effects in vitro and in vivo. MECOM, in turn, transcriptionally activates the critical oncogene KRAS, and this axis can be disrupted by JIB-04’s epigenetic modulation of MECOM. Collectively, our findings highlight MECOM as a therapeutically actionable oncogene in ovarian cancer and demonstrate that small-molecule inhibitors like JIB-04, by epigenetically targeting amplified oncogenes, offer a promising strategy for ovarian cancer treatment.

## Materials and methods

### Cell culture

A2780 ovarian cancer cells were obtained from European Collection of Authenticated Cell Cultures (ECACC) (Sigma catalog no. 93112519), and SKOV3 cells were purchased from National Cell Line Repository, National Centre for Cell Science (NCCS), Pune, India. OVSAHO cells were a kind gift from Dr. Anirban Mitra, Indiana University School of Medicine, Bloomington, IN. BJ-TERT cell line was a kind gift of Prof. Stephen Jackson, Gurdon Institute, University of Cambridge, UK. All the cell lines were tested for absence of mycoplasma every 6 months using DAPI staining. A2780 and OVSAHO cells were cultured in RPMI 1640 media (HiMedia Laboratories LLC), while SKOV3 and BJ-TERT in DMEM (HiMedia Laboratories LLC) culture media. RPMI 1640 and DMEM were supplemented with 10% fetal bovine serum (FBS, Gibco), 100 U/mL penicillin, and 100 µg/mL streptomycin (HiMedia Laboratories LLC). Cells were grown in CO_2_ incubator with optimal environment of 37 °C and 5% CO_2_ in a humidified atmosphere.

### Cell viability assay

Ovarian cancer cells were seeded in 96-well plates at different cellular densities, i.e., 10,000 cells/well for OVSAHO and A2780, while 3000 cells/well for SKOV3. After 24 h, when the cell confluency reaches to 30-40%, cells were treated with varying concentrations of small-molecule epigenetic inhibitors alone or in combination with cisplatin. Untreated cells were treated with PBS in case of cisplatin and with 1% DMSO in case of inhibitor, as vehicle control. The library of small-molecule epigenetic inhibitors used was GSK-J4 (Sigma SML0701), PFI-2, LLY-507, SGC0946, GSK343, all obtained from Structural Genomics Consortium (SGC), UK, Z-JIB-04 (Selleckchem, S0201), and E-JIB-04 (Medchem Express HY-13953). Cells were incubated with respective epidrugs/chemical probes for 72 h, and thereafter the plates were washed with 1x Phosphate Buffered Saline (PBS). The plates were then stained with 0.5% crystal violet (SRL Chemicals) made in 20% methanol solution for 20 min at RT. After staining, the plates were rinsed in distilled water and dried overnight at RT. To solubilize the stained cell biomass, 100–150 µl of methanol was added for 20 min at RT. The optical density (OD) of the plates was measured at 570 nm using microplate reader (Thermo Fisher Scientific). The background signal was subtracted from the readings of treated and control wells, and percentage cell viability was calculated using the following equation:

$$\mathrm{Cell\; viability}\, \% =(\mathrm{Absorbance\; of\; treated\; well}/\mathrm{Absorbance\; of\; control\; well})* 100$$

### siRNA transfection

For transient knockdown of *MECOM*, cells were transfected with *MECOM* specific short interfering RNA (siRNA) oligonucleotides using Lipofectamine RNAiMAX (Invitrogen) as per manufacturer’s protocol. RNA and protein were extracted post 72 h of transfection. For negative control, siRNA targeting Luciferase (siLuc) was used. MECOM targeting siRNA was commercially procured from Ambion (catalogue no. s4872) while siLuc was purchased from Eurofins with a sequence of sense 5′- > 3′ CGUACGCGGAAUACUUGCA and antisense 5′- > 3′UGCAAGUAUUCCGCGUACG.

### Cell cycle analysis

Cells were seeded to reach a confluency of 30–40% in 6 cm cell culture dish and were treated with relevant inhibitor and further incubated for 48–72 h. Cells were trypsinised and then centrifuged at 2000rpm for 10 min. Ice cold PBS was used to wash the cells, and they were fixed in 70% ethanol overnight at −20 °C. Next day, ethanol-fixed cells were centrifuged at 11,000 rpm for 5 min and resuspended in PBS. Cells were stained with propidium iodide stain (50 μg/mL) (SRL Chemicals) for 15 min at room temperature (RT) along with 100 μg/mL RNase A, and cell cycle was analysed using flow cytometry (BD Accuri C6 flow cytometer; BD Biosciences).

### Transwell migration assay

Transwell migration assay was performed using 8.0 µm transwell hanging inserts (SPL Life Sciences) in 24-well cell culture plates. 2 × 10^4^ cells were seeded on the insert in appropriate media lacking FBS, while the bottom was filled with complete media with 10% FBS as a chemoattractant. After 24 h of incubation, the migrated cells were fixed using 4% paraformaldehyde (PFA) and stained with 0.2% crystal violet solution for 30 min. After overnight drying, the images were taken for stained cells, and the percentage migration of migrated cells was quantified by dissolving crystal violet stain with methanol, and absorbance was taken at 570 nm.

### Colony formation assay

1000–2000 cells were seeded in 6-well plates and treated with indicated drugs/siRNA post 24 h of seeding. The cells were then allowed to form colonies for 14 days. Plates were then washed by 1x phosphate buffer saline, stained with 0.5% crystal violet for 30 min and washed with water and air-dried. As SKOV3 cells did not form distinct colonies, hence, percentage survival fraction of the treated and untreated cells was quantified by dissolving the stained cells in methanol and absorbance was taken at 570 nm.

### Real-time Quantitative Reverse Transcription PCR (qRT-PCR)

RNeasy® RNA isolation kit (Qiagen) was used to isolate total RNA from the cells as per the manufacturer’s instructions, and the extracted RNA was kept at −80 °C until required. Isolated RNA was treated with DNase (TURBO DNA-free kit; Invitrogen) to remove DNA contamination. RNA was quantified using nano-drop spectrophotometer (Thermo Multiskan), and RNA integrity was ensured by performing agarose gel electrophoresis. One microgram total RNA was reverse transcribed to cDNA using RevertAid First Strand cDNA Synthesis Kit as per the manufacturer’s protocol. qRT-PCR was performed for quantifying gene expression using the real-time PCR machine (The StepOnePlus™ Real Time System; Applied Biosystem). Glyceraldehyde 3-phosphate dehydrogenase (GAPDH) was used as control to normalize and calculate relative gene expression by ΔCt method, and fold change in expression was quantified as 2^−ΔΔCT^. The primer sequences used in the study are provided in Supplementary Table 1.

### Immunoblotting

Cellular protein was extracted using CSK extraction buffer (10 mM PIPES-pH 6.8; 0.3 M NaCl; 0.3 M Sucrose; 3 mM MgCl_2_; 1 mM EGTA; 0.1% Triton-X100) supplemented with proteinase inhibitor cocktails (Roche® Life Science) and phosphatase inhibitors (Roche® Life Science) after requisite treatments. Protein concentration was determined by bicinchoninic acid (BCA) method using Pierce BCA Protein Assay Kit (Thermo Fisher Scientific) as per the given instructions. Equal concentrations of extracted proteins were electrophoresed on SDS-PAGE and transferred to nitrocellulose membrane. The membrane was blocked using 3% BSA in 1x Tris-buffered saline containing 0.1% Tween-20 (1x TBST) and thereafter incubated with primary antibody against the targeted protein/histone modification and HRP-conjugated secondary anti-rabbit or anti-mouse antibodies. The protein bands were detected using Enhanced Chemiluminescence (ECL) solution (Clarity^TM^ BioRad Laboratories) and imaged by GE ImageQuant LAS 500 Imager. Densitometric analysis of protein bands was performed by ImageJ software (NIH, Bethesda, MD, USA). Supplementary Table 2 enlists the antibodies used in this study.

### Immunofluorescence

SKOV3 cells (5 × 104) were seeded on coverslips in 24 well plate and treated with E-JIB-04 for 72 h. Control cells were treated with 1% DMSO as vehicle. After 72 h of treatment, cells were fixed with 4% paraformaldehyde for 10 min at room temperature. The coverslips were then washed with PBS three times and permeabilized with 0.1% triton X-100 in PBS. The coverslips were blocked with 5% bovine serum albumin (BSA) in PBS containing 0.1% Tween-20 (PBST) for 1 h followed by incubation with mouse anti-human α-tubulin antibodies (1:1000) diluted in blocking buffer for 1 h at RT. Cells were washed thrice with PBST and incubated with Alexa Fluor^TM^ 594 goat anti-mouse IgG (Invitrogen) (1:1000) and counter-stained with 4′,6-diamidino-2-phenylindole (DAPI) (Sigma). The stained coverslips were mounted on glass slides and were imaged using Nikon A1 confocal microscope.

### Chromatin immunoprecipitation (ChIP)

Chromatin immunoprecipitation (ChIP) was performed as described [79]with minor modifications. SKOV3 cells (1 × 107) treated either with E-JIB-04 or with 1% DMSO as vehicle control, were crosslinked with 1% formaldehyde, and the reaction was stopped using 0.125 M glycine, and chromatin was extracted. Crosslinked chromatin was sonicated to generate DNA fragments of 200–700 bp using cup-horn ultrasonic processor (Antylia Scientific). The sonicated chromatin was immunoprecipitated with indicated antibodies overnight at 4 °C. Immunoprecipitation using ChIP-grade MECOM antibody was done to analyse its binding to *KRAS* promoter. Normal rabbit Immunoglobulin G (Rabbit IgG) was used as negative control. Details of all the ChIP-grade antibodies used for this study are presented in Supplementary Table 2. All the samples were reverse cross-linked, and the purified DNA was used for quantitative PCR. The ChIP-qPCR primers designed specific for *MECOM* and *KRAS* promoter/exonic regions are provided in Supplementary Table 3. The relative enrichment was calculated using fold enrichment method using rabbit IgG antibodies as the negative control of three independent experiments, plotted as mean ± SEM.

### In vivo study in SCID mice

Animal experimentation was carried out in female SCID mice, and studies reported in the present article were approved by the Institutional Animal Ethics Committee (IAEC), and all animal experiments were carried out in strict compliance with the institutional guidelines following the relevant national laws related to the conduct of animal experimentation. Cell line-derived xenograft model was established as follows. Approximately 5 × 10^6^ SKOV3 cells in 100 µL DMEM medium were subcutaneously injected into the left flank of female SCID mice (6–8 weeks, *n* = 6). Palpable tumors were observed ~20 days after induction. Tumor dimensions (mm) were measured using vernier calipers, and the volume of tumor was calculated using the formula *V* = length × (width)² × 0.5. Mice were randomized by simple randomization into 2 groups, each comprising of 3 mice i.e., control group (DMSO as vehicle) and treatment group (50 mg/kg E-JIB-04). Blinding was not implemented in this study. To minimize bias, standardized protocols were strictly followed. Additionally, two independent researchers verified the results to ensure objectivity. The mice were intratumorally injected weekly once with the drug for 3 weeks (0th, 7th, and 14th day). Tumor dimensions were measured on alternate days, and tumor volume was calculated and plotted as mean ± SD. Statistical significance was measured using one-tailed paired *t* test considering *p* value < 0.05 as statistically significant. Animals were sacrificed after 21 days of inhibitor administration. Tumors from both groups were excised and imaged. The tumor tissues were snap frozen in liquid nitrogen, and RNA was extracted to analyse target genes.

### RNA sequencing and data analysis

Total RNA was extracted using RNeasy® RNA isolation kit (Qiagen) following the manufacturer’s protocol. DNase treatment of RNA was done using TURBO DNA-free kit, Invitrogen to ensure purity. Further concentration and purity of RNA were checked using Qubit4 (Thermofisher). Two replicates from each group (DMSO or JIB-04-treated OVSAHO cells; siLuc or siMECOM-transfected OVSAHO cells) were used for library preparation. The sequencing libraries were produced using SMARTer Stranded RNA-seq kit (Takara) following the manufacturer’s instructions. The RNA library sequencing was performed by Medgenome Labs Ltd, Bangaluru, India on Illumina HiSeq X system.

The quality assessment of raw sequencing data was performed using FastQC (https://www.bioinformatics.babraham.ac.uk/projects/fastqc/) to ensure data integrity. Paired-end raw reads were trimmed quality trimming with Trimmomatic, To removed low-quality bases and adapter sequences, paired-end raw reads were trimmed using Trimmomatic, ensuring high-quality data for downstream analysis. Quality-trimmed paired-end RNA-Seq reads were aligned to the Homo sapiens GRCh38 reference genome using HISAT2, with the reference genome indexed via hisat2-build [80]. SAM files generated from alignment were converted to BAM files for further sorting and indexing using SAM tools. These sorted BAM files were used as input for HTSeq-count to quantify gene expression levels, employing Homo_sapiens.GRCh38.112.gtf.gz for gene annotation. Subsequently, RNA-seq data analysis was conducted using edgeR to identify differentially expressed genes (DEGs) [81]. Lowly expressed genes were filtered, and normalization was applied using the TMM (trimmed mean of M-values) method. Log-transformed counts per million (logCPM) values were calculated, and a principal component analysis (PCA) was performed for sample clustering. Gene-wise dispersion was estimated, and a quasi-likelihood F-test was used to detect DEGs. The results were visualized using MA and volcano plots based on *P* value thresholds <0.05. Z-scores for DEGs were computed and capped between −2 and +2, followed by hierarchical clustering and heatmap generation with row and column annotations using Complex Heatmap. This analysis explored gene expression patterns across different samples and highlighted distinct clusters of genes.

Gene Ontology (GO) analysis of DEGs was performed separately for upregulated and downregulated genes using the web version of DAVID [82, 83]. Bar and Dot plots with a *P* value threshold of less than 0.05 were generated using ggplot-2 and dplyr to visualize the ontology graphs, providing insights into the biological functions and pathways associated with the DEGs. From both the experiments up and down regulated genes were split, and Venn diagram was made using Bioconductor package vennDiagram and grid to visualize and interpret common genes in both the experimental conditions [84]. The RNA sequencing raw data is submitted to NCBI BioProject accession number PRJNA1184709.

### Public dataset-based analysis

The publicly available datasets profiling copy number amplification of genes in tumor samples of cancer patients were analysed using cBioportal (http://cbioportal.org) web portal [36, 37]. Further, transcriptional expression of MECOM in various tumors and their corresponding normal tissue samples was analysed using GENT2A database (http://gent2.appex.kr/gent2/) [38]. MECOM expression in ovarian cancer datasets GSE14407 [39] and GSE118520 [40]was analysed using GEO2R data analysis tool. Both of the datasets are based on the GPL570 platform (HG-U133_Plus_2; Affymetrix Human Genome U133 Plus 2.0 Array). The web-based Gene_Corr module of TIMER2.0 webserver (http://timer.comp-genomics.org/) was used to analyse correlation of gene expression in ovarian cancer TCGA database [59].

### Statistical analysis

All data are mean of minimum of three independent experiments ± standard deviation (mean ± SD) unless otherwise stated. All statistical analyses were carried out using software Prism 5 for Windows (GraphPad Prism 5). The *p* values were determined using student’s unpaired *t-*test considering *p* value < 0.05 as statistically significant unless otherwise indicated. Other statistical analysis, such as Mann–Whitney U test, Spearman’s Rank-Order Correlation were used as appropriate.

## Supplementary information

Supplementary data

WB uncropped file

## Data Availability

All data generated or analysed during this study are included in this article.
