# Supplementary material for: Epigenetic targeting of MECOM/KRAS axis by JIB-04 impairs tumorigenesis and cisplatin resistance in MECOM-amplified ovarian cancer
Source: Cell Death Discov. 2025 Jul 15;11:326. doi: 10.1038/s41420-025-02618-2 (PMC12264112; doi:10.1038/s41420-025-02618-2)
Supplement: Supplementary file 1 — Supplementary data [file 41420_2025_2618_MOESM1_ESM.docx]

**Supplementary Information:
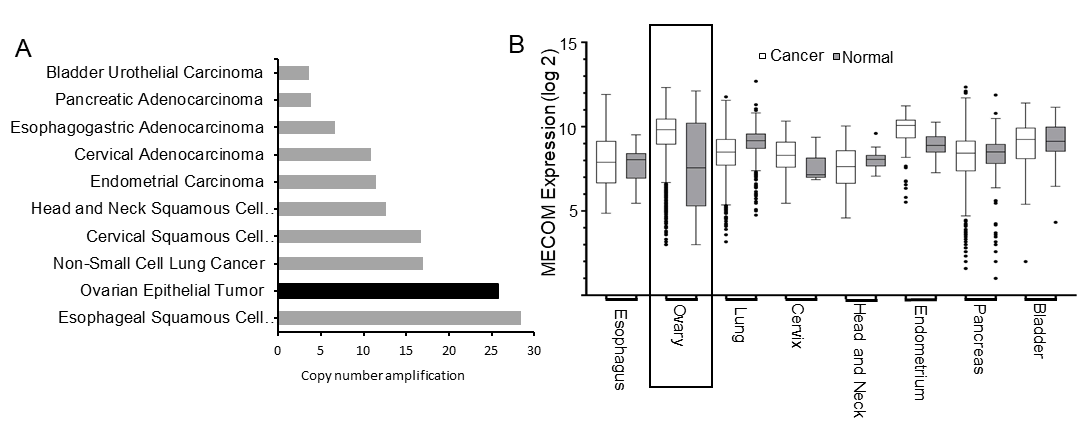
**

**Supplementary figure 1: MECOM is amplified and overexpressed in ovarian cancer patient database.** (A) TCGA data of cancers with high copy number amplification of MECOM (>5%) (B) Mean log2 mRNA expression of MECOM in tumors paired with normal tissues for various cancers analysed by GENT2 gene expression platform

**Supplementary figure 2: Effect of JIB-04 pan-histone demethylase inhibitor on cell viability of non-tumorigenic BJ-TERT cells and activity test by analysing histone methylation marks.** (A) No growth inhibitory effect was observed on non-tumorogenic cell BJ-TERT fibroblasts. (B&C) Western blotting demonstrated increase in H3K27me3, H3K9me3 and H3K4me3 methylation histone marks in E-JIB-04 and Z-JIB-04 treated SKOV3 ovarian cancer cells.

**
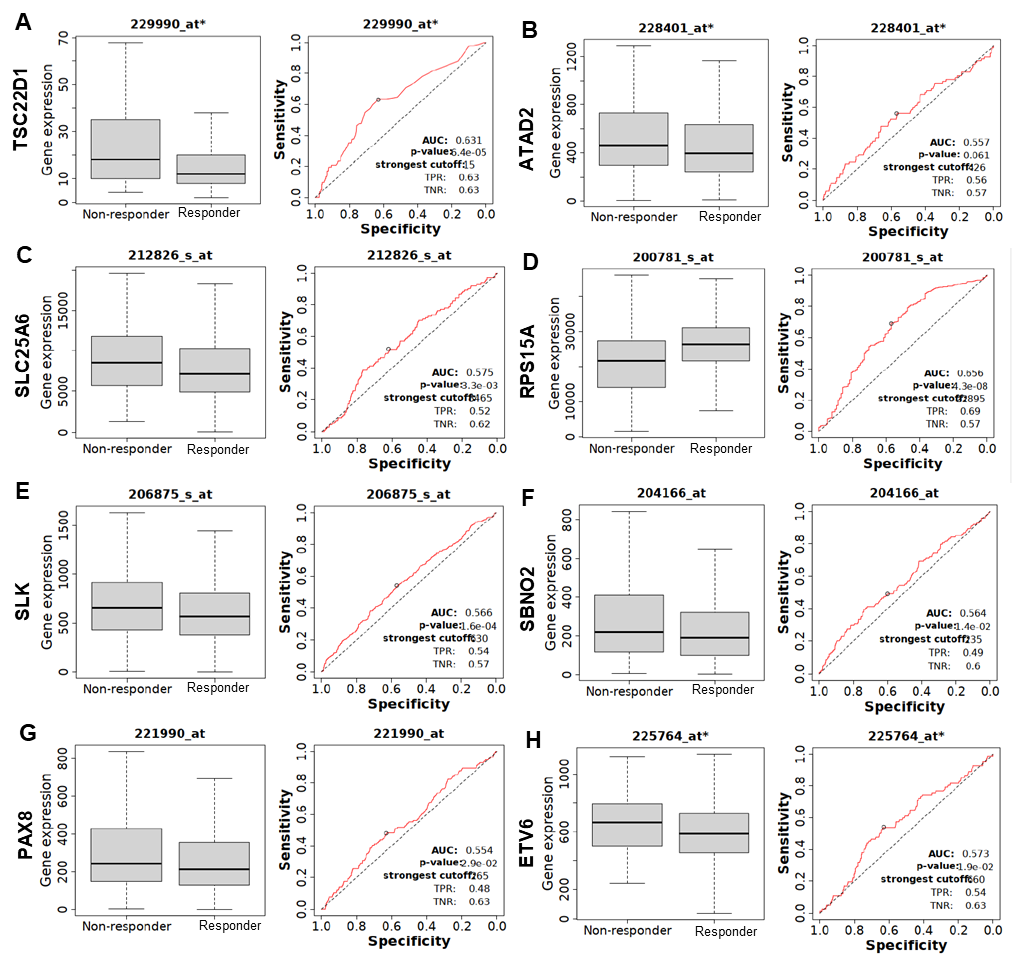
**

**Supplementary figure 3: ROC plot analysis of common differentially expressed genes.** (A – H) Expression of patients who are not responding to platin drug and known as non-responders (n =114) were compared to responders (n =1095). ROC curves demonstrate correlation of common genes with AUC>0.55 with platinum resistance. Ovarian cancer patient cohorts were treated with platin drugs and the response outcomes were determined by relapse free survival over 6 months.

**Supplementary Tables:**

**Supplementary Table 1: List of qRT-PCR primers.**

| **Gene target** | **Forward Primer (5’- 3’)** | **Reverse Primer (3’-5’)** |
| --- | --- | --- |
| GAPDH | CTTTGGTATCGTGGAAGGACTC | GTAGAGGCAGGGATGATGTTC |
| MECOM | CCAGGAATGTGGAGGAGAGA | CCTCCTCATCTAACAACACCTCA |
| KRAS | GACTCTGAAGATGTACCTATGGTCCTA | CATCATCAACACCCTGTCTTGTC |
| PIK3CA | CCACGACCATCATCAGGTGAA | CCTCACGGAGGCATTCTAAGT |
| GADD45A | TGTGCTGGTGACGAACC | ACCCACTGATCCATGTAGCG |
| GADD45B | GTCGGCCAAGTTGATGAAT | CACGATGTTGATGTCGTTGT |
| EGR1 | ATTGATGTCTCCGCTGCAGATC | TCAGCAGCATCATCTCCTCCA |
| ZEB1 | AGGATGACCTGCCAACAGAC | CTTCAGGCCCCAGGATTTCTT |
| Vimentin | CTGCCAACCGGAACAATGAC | CATTTCACGCATCTGGCGTT |
| Fibronectin | TGGGCAACTCTGTCAACGAA | CCACTCATCTCCAACGGCAT |
| N-Cadherine | GTGCATGAAGGACAGCCTCT | CCACCTTAAAATCTGCAGGC |

**Supplementary Table 2: List of antibodies used in this study.**

| **Antibody** | **Species** | **Application** | **Reference/ Company** |
| --- | --- | --- | --- |
| EVI-1 (C50E12) | Rabbit | WB, ChIP | Cell Signalling Technology (2593S) |
| Actin | Rabbit | WB | Sigma (A2066) |
| Tubulin | Mouse | WB/IF | Sigma (T9026) |
| GAPDH (14C10) | Rabbit | WB | Cell Signalling Technology (2118S) |
| H3K27me3 | Rabbit | WB, ChIP | Abcam (ab192985) |
| H3K4me3 | Rabbit | WB, ChIP | Abcam (ab8580) |
| H3K9me3 | Rabbit | WB, ChIP | Abcam (ab8898) |
| ERK1/2 | Rabbit | WB | Cell Signalling Technology (4695T) |
| pERK/2 | Rabbit | WB | Cell Signalling Technology (4370T) |
| Goat anti-Rabbit IgG (H+L) Cross-Adsorbed Secondary Antibody, HRP | Rabbit | WB | Thermo Fischer Scientific (G21234) |
| Goat anti-Mouse IgG (H+L) Secondary Antibody, HRP | Mouse | WB | Invitrogen (31430) |

**Supplementary Table 3: List of ChIP - qPCR primers used in this study.**

| **ChIP qPCR Primers** | **Forward Primer (5’- 3’)** | **Reverse Primer (3’-5’)** | **Localisation from**  **TSS** |
| --- | --- | --- | --- |
| MECOM-Primer 1 | CCTTGTACACAGCTAGCCCC | ACAACCTCGGGGTCAAAAGA | -820 to -700 |
| MECOM-Primer 2 | CCGACCTCTGAGAAAGCAGG | AATGCTTGCTGCCATCCTTG | -600 to -510 |
| MECOM-Primer 3 | TTCAGTAGCAGTAGCAGCGG | GAACTAGAAGGGCTGGACCC | -390 to -300 |
| MECOM-Primer 4 | ATCAATCCTCTGCCCAAGCC | TCAGATGGCAATCGCCGAG | -120 to 0 |
| MECOM-Primer 5 | CTCGGCGATTGCCATCTGA | CTAGACGCCCCTCCAACATC | 0 to +90 |
| KRAS-Primer 1 | TCAAGTGGAATGAGGGCCAC | ACACGGGCTGTAATCTGCAA | -1540 to -1210 |
| KRAS-Primer 2 | TTGCAGATTACAGCCCGTGT | AGCGCCTGTACCTGATAGGA | -1230 to -1050 |
| KRAS-Primer 3 | TCCTATCAGGTACAGGCGCT | GATCGCCGCCCCGATTATTA | -1070 to -620 |
| KRAS-Primer 4 | GTCGTGTTTGTTTGGGCCTG | CGGTCTCCACAGAGAAGCTG | -700 to -450 |

**Supplementary Table 4: List of small molecule epigenetic inhibitors screening in this study.**

| **Epigenetic Inhibitor** | **Targets** | **Histone marks affected** |
| --- | --- | --- |
| E-JIB04/Z-JIB-04(1) | Histone demethylase KDM5A/6B/4A-E | H3K4me3/H3K27me3/H3K9me3 |
| GSK-J4(2) | Histone demethylase KDM6B | H3K27me3 |
| SGC0946(3) | Histone methyltransferase DOT1L | H3K79me2 |
| GSK343(4) | Histone methyltransferase EZH2 | H3K27me3 |
| PFI-2(5) | Histone methyltransferase SETD7 | H3K4me1 |
| LLY507(6) | Histone methyltransferase SMYD2 | H3K4me3 |

**Supplementary Table 5: List of Upregulated Genes Overlapped Between JIB-04 Treated Cells and siMECOM Cells**

| **Upregulated Genes** | | |
| --- | --- | --- |
| **S.No.** | **Ensemble id** | **Gene name** |
|  | ENSG00000096696 | Desmoplakin(DSP) |
|  | ENSG00000064932 | strawberry notch homolog 2(SBNO2) |
|  | ENSG00000065613 | STE20 like kinase(SLK) |
|  | ENSG00000004799 | Pyruvate Dehydrogenase Kinase 4(PDK4) |
|  | ENSG00000102804 | TSC22 domain family member 1(TSC22D1) |
|  | ENSG00000113580 | Nuclear Receptor Subfamily 3 group C member 1(NR3C1) |
|  | ENSG00000118503 | TNF alpha induced protein 3(TNFAIP3) |
|  | ENSG00000125618 | Paired box 8(PAX8) |
|  | ENSG00000139083 | ETS Variant Transcription Factor 6(ETV6) |
|  | ENSG00000140575 | IQ motif containing GTPase activating protein 1(IQGAP1) |
|  | ENSG00000143631 | Filaggrin(FLG) |
|  | ENSG00000146072 | TNF Receptor Superfamily Member 21(TNFRSF21) |
|  | ENSG00000152402 | Guanylate Cyclase 1 Soluble Subunit Alpha 2(GUCY1A2) |
|  | ENSG00000157106 | SMG1 nonsense mediated mRNA decay associated PI3K related kinase(SMG1) |
|  | ENSG00000169100 | Solute Carrier Family 25 member 6(SLC25A6) |
|  | ENSG00000181143 | Mucin 16, Cell Surface associated(MUC16) |
|  | ENSG00000198743 | Solute Carrier Family 5 member 3(SLC5A3) |
|  | ENSG00000265972 | Thioredoxin interacting protein (TXNIP) |

**Supplementary Table 6: List of Downregulated Genes Overlapped Between JIB-04 Treated Cells and siMECOM Cells**

| **Downregulated Genes** | | |
| --- | --- | --- |
| **S.No.** | **Ensemble id** | **Gene name** |
|  | ENSG00000036257 | Cullin 3(CUL3) |
|  | ENSG00000074181 | Notch receptor 3(NOTCH3) |
|  | ENSG00000085276 | MDS1 and EVI1 complex locus(MECOM) |
|  | ENSG00000113387 | SUB1 regulator of transcription(SUB1) |
|  | ENSG00000106484 | Mesoderm Specific Transcript(MEST) |
|  | ENSG00000103202 | NME/NM23 nucleoside diphosphate kinase 4(NME4) |
|  | ENSG00000120802 | Thymopoietin (TMPO) |
|  | ENSG00000182481 | Karyopherin Subunit Alpha 2(KPNA2) |
|  | ENSG00000126602 | TNF Receptor Associated Protein 1(TRAP1) |
|  | ENSG00000126803 | Heat Shock Protein Family A (Hsp70) member 2(HSPA2) |
|  | ENSG00000134419 | Ribosomal rotein S15a(RPS15A) |
|  | ENSG00000132485 | Zinc Finger RANBP2-type containing 2(ZRANB2) |
|  | ENSG00000156802 | ATPase family AAA domain containing 2(ATAD2) |
|  | ENSG00000123472 | ATP synthase mitochondrial F1 complex assembly factor 1(ATPAF1) |
|  | ENSG00000183421 | Receptor Interacting Serine/threonine Kinase 4(RIPK4) |
|  | ENSG00000275215 | RNA, 5.8S ribosomal N3(RNA5-8SN3) |

**Supplementary Table 7: The enriched gene ontology (GO) categories of 34 overlapping DEGs.**

| **GO ID** | **GO Annotation** | **Genes** |
| --- | --- | --- |
| **Biological Process** | | |
| GO:0045944 | Positive regulation of transcription by RNA polymerase II | NOTCH3, SBNO2, TSC22D1, PAX8, MECOM, NR3C1, ETV6 |
| GO:0006915 | Apoptotic process | SLK, MECOM, TNFAIP3, NR3C1, TNFRSF21, SLC25A6 |
| GO:0000122 | Negative regulation of transcription by RNA polymerase II | NOTCH3, CUL3, TXNIP, NR3C1, ETV6 |
| GO:0030216 | Keratinocyte differentiation | FLG, DSP, TXNIP |
| GO:0016477 | Cell migration | CUL3, TNFAIP3, IQGAP1 |
| GO:0018149 | Peptide cross-linking | FLG, DSP |
| GO:0043069 | Negative regulation of programmed cell death | TSC22D1, MECOM |
| GO:0071425 | Hematopoietic stem cell proliferation | MECOM, ETV6 |
| GO:0048008 | Platelet-derived growth factor receptor signaling pathway | TXNIP, IQGAP1 |
| GO:0043588 | Skin development | DSP, RIPK4 |
| GO:1902895 | Positive regulation of miRNA transcription | NOTCH3, NR3C1 |
| **Cellular Component** | | |
| GO:0005634 | Nucleus | FLG, DSP, SMG1, TSC22D1, CUL3, ATAD2, TNFAIP3, HSPA2, IQGAP1, NR3C1, ETV6, SBNO2, PAX8, MECOM, SUB1, KPNA2, SLC25A6, TMPO |
| GO:0005829 | Cytosol | FLG, GUCY1A2, NOTCH3, SMG1, TSC22D1, CUL3, TNFAIP3, HSPA2, IQGAP1, NR3C1, ETV6, RPS15A, SLK, MECOM, TXNIP, KPNA2 |
| GO:0016020 | Membrane | TRAP1, MUC16, CUL3, RIPK4, HSPA2, NR3C1, SLC5A3, ETV6, RPS15A, KPNA2, MEST, TNFRSF21, SLC25A6, TMPO |
| GO:0005737 | Cytoplasm | DSP, SMG1, TSC22D1, CUL3, RIPK4, TNFAIP3, HSPA2, IQGAP1, NR3C1, RPS15A, SLK, TXNIP, KPNA2, TMPO |
| GO:0005654 | Nucleoplasm | TRAP1, NOTCH3, SMG1, RPS15A, ZRANB2, PAX8, MECOM, CUL3, SUB1, ATAD2, NR3C1, KPNA2 |
| GO:0070062 | Extracellular exosome | DSP, RPS15A, SLK, MUC16, CUL3, SUB1, ATAD2, TNFAIP3, HSPA2, IQGAP1, MEST |
| GO:0005739 | Mitochondrion | TRAP1, TSC22D1, PDK4, NME4, NR3C1, ATPAF1, SLC25A6 |
| GO:0005759 | Mitochondrial matrix | TRAP1, PDK4, NME4, NR3C1 |
| GO:0005743 | Mitochondrial inner membrane | TRAP1, NME4, ATPAF1, SLC25A6 |
| GO:0016323 | Basolateral plasma membrane | DSP, IQGAP1, SLC5A3 |
| GO:0072354 | Cornified envelope | FLG, DSP |
| **Molecular Function** | | |
| GO:0005515 | Protein binding | FLG, NOTCH3, SMG1, CUL3, TNFAIP3, IQGAP1, NR3C1, SLC5A3, RPS15A, SLK, MECOM, PDK4, KPNA2, MEST, TMPO, DSP, TRAP1, GUCY1A2, ZRANB2, MUC16, TSC22D1, RIPK4, ATAD2, NME4, HSPA2, ETV6, PAX8, SUB1, TXNIP, TNFRSF21, SLC25A6, ATPAF1 |
| GO:0003723 | RNA binding | DSP, TRAP1, SMG1, RPS15A, ZRANB2, SUB1, NR3C1, KPNA2 |
| GO:0005524 | ATP binding | TRAP1, SMG1, SLK, ATAD2, RIPK4, PDK4, NME4, HSPA2 |
| GO:0042802 | Identical protein binding | NOTCH3, SLK, TSC22D1, CUL3, SUB1, TNFAIP3, NR3C1 |
| GO:0000978 | RNA polymerase II cis-regulatory region sequence-specific DNA binding | TSC22D1, PAX8, MECOM, SUB1, NR3C1, ETV6 |
| GO:0003677 | DNA binding | PAX8, MECOM, SUB1, TNFAIP3, NR3C1, TMPO |
| GO:0001228 | DNA-binding transcription activator activity, RNA polymerase II-specific | TSC22D1, PAX8, MECOM, NR3C1, ETV6 |
| GO:0045296 | Cadherin binding | NOTCH3, SLK, IQGAP1, TMPO |
| GO:0003700 | DNA-binding transcription factor activity | PAX8, MECOM, NR3C1, ETV6 |
| GO:0072354 | Histone H3T3 kinase activity | SMG1, SLK, RIPK4 |
| GO:0004694 | Eukaryotic translation initiation factor 2alpha kinase activity | SMG1, SLK, RIPK4 |
| GO:0004711 | Ribosomal protein S6 kinase activity | SMG1, SLK, RIPK4 |
| GO:0072518 | Rho-dependent protein serine/threonine kinase activity | SMG1, SLK, RIPK4 |
| GO:0004676 | 3-phosphoinositide-dependent protein kinase activity | SMG1, SLK, RIPK4 |
| GO:0004677 | DNA-dependent protein kinase activity | SMG1, SLK, RIPK4 |
| GO:0004679 | AMP-activated protein kinase activity | SMG1, SLK, RIPK4 |
| GO:0140662 | ATP-dependent protein folding chaperone | TRAP1, HSPA2 |

**Supplementary References**

1. Wang L, Chang J, Varghese D, Dellinger M, Kumar S, Best AM, et al. A small molecule modulates Jumonji histone demethylase activity and selectively inhibits cancer growth. Nat Commun. 2013;4(2035):1–13.

2. Yan N, Xu L, Wu X, Zhang L, Fei X, Cao Y, et al. GSKJ4, an H3K27me3 demethylase inhibitor, effectively suppresses the breast cancer stem cells. Exp Cell Res. 2017 Oct 15;359(2):405–14.

3. Wong M, Polly P, Liu T. The histone methyltransferase DOT1L: regulatory functions and a cancer therapy target [Internet]. Vol. 5, Am J Cancer Res. 2015. Available from: www.ajcr.us/ISSN:2156-6976/ajcr0014308

4. Liu TP, Lo HL, Wei LS, Hsiao HHY, Yang PM. S-adenosyl-L-methionine-competitive inhibitors of the histone methyltransferase EZH2 induce autophagy and enhance drug sensitivity in cancer cells. Anticancer Drugs. 2015;26(2):139–47.

5. Barsyte-Lovejoy D, Li F, Oudhoff MJ, Tatlock JH, Dong A, Zeng H, et al. (R)-PFI-2 is a potent and selective inhibitor of SETD7 methyltransferase activity in cells. Proc Natl Acad Sci U S A. 2014 Sep 2;111(35):12853–8.

6. Nguyen H, Allali-Hassani A, Antonysamy S, Chang S, Chen LH, Curtis C, et al. LLY-507, a cell-active, potent, and selective inhibitor of protein-lysine methyltransferase SMYD2. Journal of Biological Chemistry. 2015 May 29;290(22):13641–53.
