# Supplementary figures and images for: Epigenetic targeting of MECOM/KRAS axis by JIB-04 impairs tumorigenesis and cisplatin resistance in MECOM-amplified ovarian cancer
Source: Cell Death Discov. 2025 Jul 15;11:326. doi: 10.1038/s41420-025-02618-2 (PMC12264112; doi:10.1038/s41420-025-02618-2)

Figure 1D

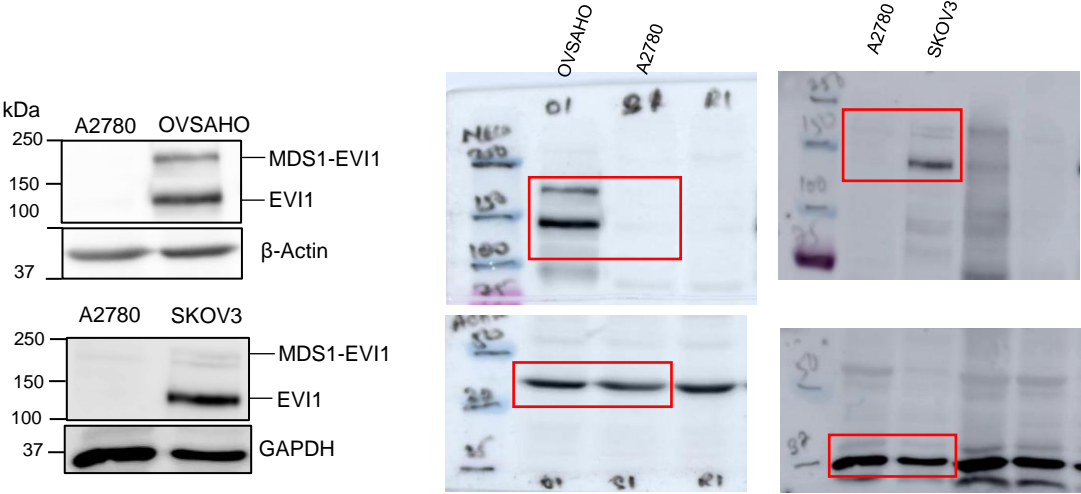

Figure 1G

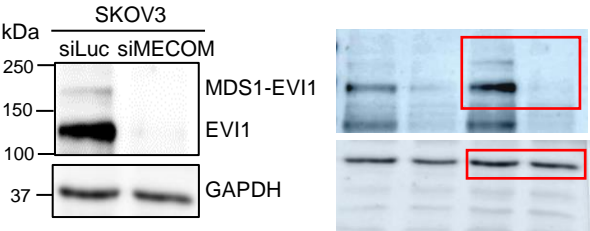

Figure 1H

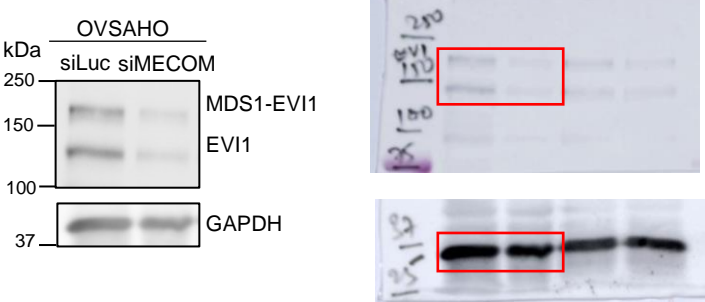

Figure 1J

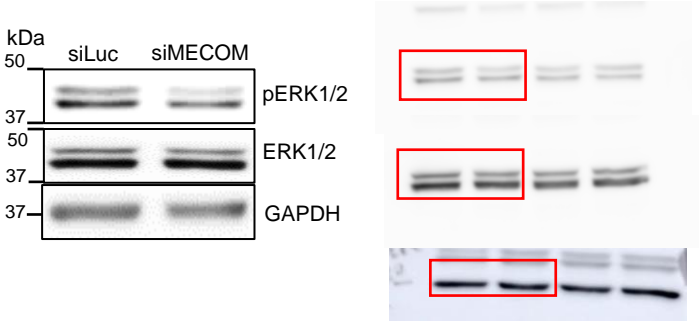

Figure 3C

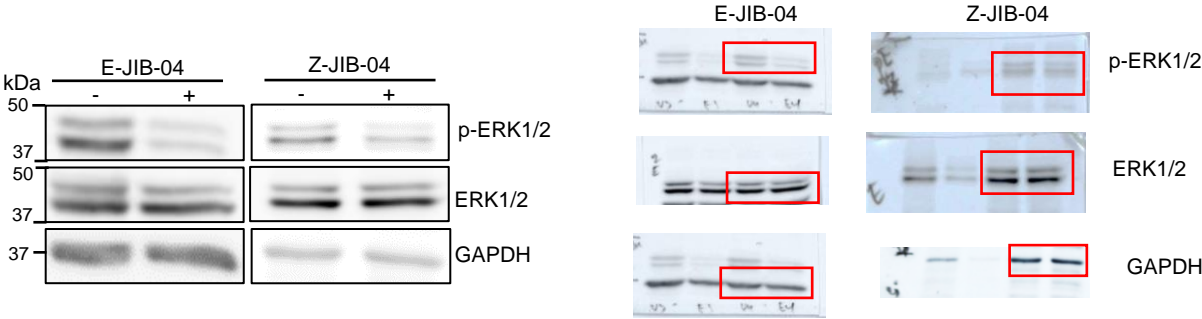

Figure 5B

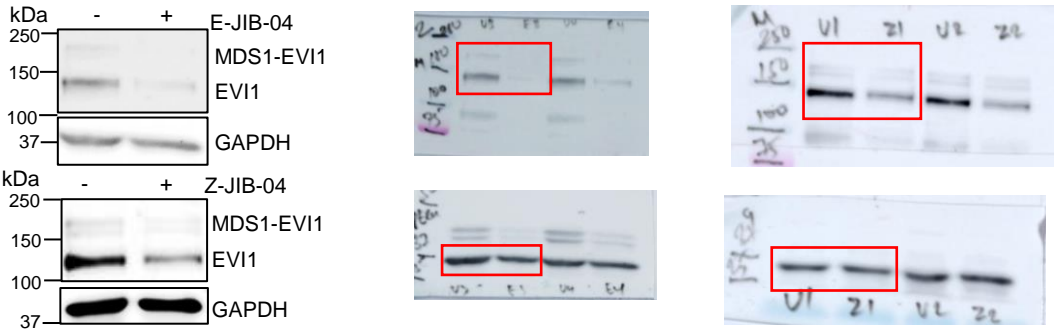

Supplement: Supplementary file 2 — WB uncropped file [file 41420_2025_2618_MOESM2_ESM.pdf]
